# Prompting and Fine-Tuning Large Language Models for Parkinson Disease Diagnosis: Comparative Evaluation Study Using the PPMI Structured Dataset

**DOI:** 10.2196/77561

**Published:** 2026-01-15

**Authors:** Hyun-Ji Shin, Young Jin Jeong, Sungmin Jun, Do-Young Kang

**Affiliations:** 1 Department of Data Sciences Convergence Graduate School Dong-A University Busan Republic of Korea; 2 Institute of Convergence Bio-Health Dong-A University Busan Republic of Korea; 3 Department of Nuclear Medicine College of Medicine Dong-A University Busan Republic of Korea; 4 Department of Nuclear Medicine Dong-A University Hospital Busan Republic of Korea

**Keywords:** Claude, diagnostic classification, fine-tuning, Gemini, GPT, large language models, LLaMA, Parkinson disease, Parkinson’s Progression Markers Initiative, PPMI, prompt engineering

## Abstract

**Background:**

Parkinson disease (PD) presents diagnostic challenges due to its heterogeneous motor and nonmotor manifestations. Traditional machine learning (ML) approaches have been evaluated on structured clinical variables. However, the diagnostic utility of large language models (LLMs) using natural language representations of structured clinical data remains underexplored.

**Objective:**

This study aimed to evaluate the diagnostic classification performance of multiple LLMs using natural language prompts derived from structured clinical data and to compare their performance with traditional ML baselines.

**Methods:**

We reformatted structured clinical variables from the Parkinson’s Progression Markers Initiative (PPMI) dataset into natural language prompts and used them as inputs for several LLMs. Variables with high multicollinearity were removed, and the top 10 features were selected using Shapley additive explanations (SHAP)–based feature ranking. LLM performance was examined across few-shot prompting, dual-output prompting that additionally generated post hoc explanatory text as an exploratory component, and supervised fine-tuning. Logistic regression (LR) and support vector machine (SVM) classifiers served as ML baselines. Model performance was evaluated using *F*_1_-scores on both the test set and a temporally independent validation set (temporal validation set) of limited size, and repeated output generation was carried out to assess stability.

**Results:**

On the test set of 122 participants, LR and SVM trained on the 10 SHAP-selected clinical variables each achieved a macro-averaged *F*_1_-score of 0.960 (accuracy 0.975). LLMs receiving natural language prompts derived from the same variables reached comparable performance, with the best few-shot configurations achieving macro-averaged *F*_1_-scores of 0.987 (accuracy 0.992). In the temporal validation set of 31 participants, LR maintained a macro-averaged *F*_1_-score of 0.903, whereas SVM showed substantial performance degradation. In contrast, multiple LLMs sustained high diagnostic performance, reaching macro-averaged *F*_1_-scores up to 0.968 and high recall for PD. Repeated output generation across LLM conditions produced generally stable predictions, with rare variability observed across runs. Under dual-output prompting, diagnostic performance showed a reduction relative to few-shot prompting while remaining generally stable. Supervised fine-tuning of lightweight models improved stability and enabled GPT-4o-mini to achieve a macro-averaged *F*_1_-score of 0.987 on the test set, with uniformly correct predictions observed in the small temporal validation set, which should be interpreted cautiously given the limited sample size and exploratory nature of the evaluation.

**Conclusions:**

This study provides an exploratory benchmark of how modern LLMs process structured clinical variables in natural language form. While several models achieved diagnostic performance comparable to LR across both the test and temporal validation datasets, their outputs were sensitive to prompting formats, model choice, and class distributions. Occasional variability across repeated output generations reflected the stochastic nature of LLMs, and lightweight models required supervised fine-tuning for stable generalization. These findings highlight the capabilities and limitations of current LLMs in handling tabular clinical information and underscore the need for cautious application and further investigation.

## Introduction

Parkinson disease (PD) is the second most common neurodegenerative disorder and presents substantial diagnostic challenges due to the heterogeneity of its motor and nonmotor symptoms [1-3]. Despite sustained research efforts, early diagnostic accuracy remains limited, and the variability of clinical presentations continues to complicate reliable classification [4,5]. Large-scale initiatives such as the Parkinson’s Progression Markers Initiative (PPMI) [6] have facilitated systematic evaluation of clinical, imaging, and biomarker variables, providing a foundation for quantitative approaches to PD diagnosis.

Machine learning (ML) models, including logistic regression (LR) [7], support vector machines (SVM) [8], and tree-based classifiers, have demonstrated strong performance when applied to structured clinical variables [9-11]. However, these models operate on fixed feature representations and do not naturally support flexible natural language inputs or generative reasoning, limiting their applicability when structured clinical data must be reformatted into descriptive text.

Recent large language models (LLMs) exhibit strong capabilities in processing natural language information and have shown promise in clinical applications involving unstructured text such as clinical notes and reports [12,13]. LLM-generated rationales typically represent post hoc explanatory text rather than true interpretability [14], and prior studies have reported challenges such as prompt sensitivity, stochastic variability, and susceptibility to distributional shifts when LLMs are applied to medical tasks [15]. However, their ability to perform diagnostic classification when structured clinical variables are reformatted into natural language prompts remains insufficiently explored, and recent work evaluating the performance of LLMs on structured or tabular data has reported limited and inconsistent results [16]. These characteristics highlight the need for careful and systematic evaluation when applying LLMs to structured clinical information.

In this context, the goal of this study was to conduct an exploratory benchmark of modern LLMs for PD classification using natural language prompts derived from structured clinical variables in the PPMI dataset. We compared multiple LLM families with traditional ML baselines and evaluated their behavior across few-shot prompting, dual-output prompting, and supervised fine-tuning. The study aimed to characterize both the capabilities and the limitations of LLMs in handling tabular clinical information, rather than demonstrating superiority over conventional ML approaches.

## Methods

### Overview

**Figure 1:**
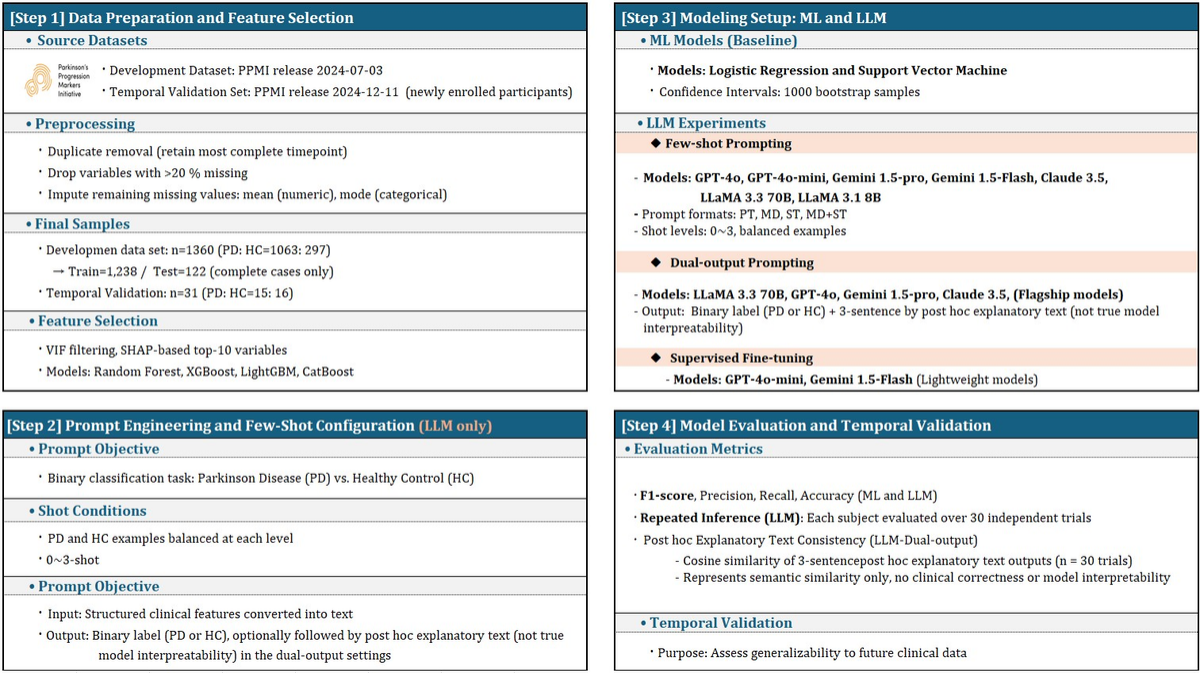
Study design overview illustrating the overall experimental pipeline, including dataset selection, prompting strategies, fine-tuning, and evaluation procedures. HC: healthy controls; LLM: large language model; MD: markdown; MD+ST: markdown with special token; ML: machine learning; PD: Parkinson disease; PPMI: Parkinson’s Progression Markers Initiative; PT: plain text; SHAP: Shapley additive explanations; ST: special token; VIF: variance inflation factor.

Figure 1 illustrates the overall methodological workflow of the study, detailing the progression from dataset selection and feature preprocessing to prompt construction, model training, and evaluation. It provides a concise visual summary of the experimental pipeline described in the Methods section.

### Dataset and Preprocessing

This study used a curated dataset provided by the PPMI, downloaded on July 29, 2024 (data release: July 3, 2024). The initial dataset consisted of 4 participant cohorts: individuals with PD, healthy controls (HC), participants with scans without evidence of dopaminergic deficit, and prodromal participants. For this study, only PD and HC participants were included.

Duplicate observations were removed by retaining the most comprehensive examination timepoint for each participant. Variables with more than 20% of missingness were excluded. Missing values in the remaining viables were imputed using mean imputation for numerical variables and mode imputation for categorical variables [17]. Participants included in the held-out test set and in the temporally independent validation set (temporal validation set) did not contain missing values and therefore required no imputation. Missing-value imputation was performed only for the training portion of the development dataset, which consisted of the train and validation subsets. Imputation statistics (mean and mode) were computed exclusively from the training split and subsequently applied to validation split. This procedure ensured that no information from the test or temporal validation sets influenced the preprocessing of the training data. After preprocessing, the final development dataset consisted of 1360 participants (PD, n=1063; HC, n=297).

A temporal validation set was constructed using the most recent curated PPMI dataset released on December 11, 2024. To ensure temporal separation, all data contained in the July 3, 2024, release were excluded, and only data from participants newly recruited after this release were included. This temporally separated dataset was used to assess the generalizability of model performance to data collected at a later timepoint. In clinical research, obtaining an external dataset with identical conditions is often challenging, and temporally separated datasets serve as a practical and widely accepted alternative for external validation [18,19].

Demographic characteristics of both the development and temporal validation datasets, including age, sex, education, and race, are summarized in Table 1.

**Table 1:** Demographic information of participants by dataset group.

Characteristic		Development set		Temporal validation set^a^	
		PD^b^ (n=1063)	HC^c^ (n=297)	PD (n=15)	HC (n=16)
Age (years), mean (SD; range)		63.3 (9.7; 30.7-85.3)	62.4 (10.6; 30.4-83.7)	62.7 (10.9; 31.3-73.0)	63.9 (11.3; 30.4-79.4)
**Sex, n (%)**					
	Male	686 (64.5)	195 (65.7)	8 (53.3)	3 (18.8)
	Female	377 (35.5)	102 (34.3)	7 (46.7)	13 (81.3)
Education (years), mean (SD; range)		16.0 (2.8; 6-20)	16.0 (2.7; 8-20)	16.8 (2.0; 11-20)	16.7 (2.0; 12-20)
**Race, n (%)**					
	White	973 (91.5)	272 (91.6)	13 (86.7)	16 (100)
	Black	30 (2.8)	10 (3.4)	0 (0)	0 (0)
	Asian	21 (2)	2 (0.7)	1 (6.7)	0 (0)
	Other	39 (3.7)	13 (4.4)	1 (6.7)	0 (0)

^a^Temporal validation set includes participants newly enrolled after the July 3, 2024, data release (excluded from the development set) and serves as a temporally separated dataset for external validation.

^b^PD: Parkinson disease.

^c^HC: healthy controls.

The development dataset was randomly divided into training, validation, and test subsets following an approximately 7:2:1 ratio. Only complete cases were included in the test set to ensure reliable evaluation. Feature selection and fine-tuning were performed exclusively using the training set. The test set was used solely for final model evaluation and was not involved in any feature selection or model fitting procedures. Class distributions for each subset are summarized in Table 2.

**Table 2:** Dataset splits and class distribution for diagnostic modeling.

Datasets and subsets		Total, n	PD^a^, n (%)	HC^b^, n (%)
**Development set**				
	Training set	990	771 (77.9)	219 (22.1)
	Validation set	248	193 (77.8)	55 (22.2)
	Test set	122	99 (81.1)	23 (18.9)
Temporal validation set		31	15 (48.4)	16 (51.6)

^a^PD: Parkinson disease.

^b^HC: healthy controls.

### Feature Selection

#### Multicollinearity Evaluation

To address multicollinearity, we generated a correlation matrix and computed the variance inflation factor for all candidate variables [20]. Variables with variance inflation factor values exceeding 10, a commonly accepted threshold for severe multicollinearity, were removed to prevent unstable coefficients and improve model reliability [21,22]. A total of 70 variables remained after this screening step and were used for Shapley additive explanations (SHAP)–based feature selection.

#### SHAP-Based Feature Importance

Feature importance was evaluated using a weighted average of SHAP values obtained from 4 tree-based models, including random forest [23], XGBoost [24], LightGBM [25], and CatBoost [26]. These models were selected because they are widely used for their tabular data and are known to mitigate issues related to multicollinearity while accommodating mixtures of numerical and categorical features effectively [27].

For each model, SHAP values were computed for all remaining variables, normalized, and then combined using a weighted averaging approach based on the diagnostic performance of each model. The performance metrics and assigned weights are summarized in Multimedia Appendix 1.

The weighted average SHAP value for each feature was calculated as [28]:


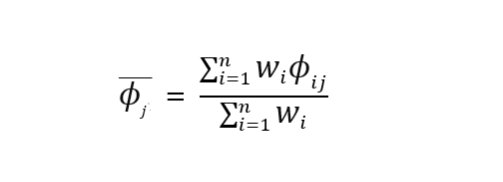


where:

- *ϕ_ij_* represents the SHAP value of feature *j* computed by model *i*
- *w_i_* represents the performance-based weight assigned to model *i*
- the denominator 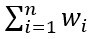 ensures that weights are normalized so they sum to 1.

The top 10 variables were selected based on these weighted average SHAP values, as shown in Multimedia Appendix 2. The final features set consisted of clinically meaningful motor, olfactory, and imaging features. Specifically, the selected variables were updrs3_score, con_putamen, updrs_totscore, updrs2_score, lowput_expected, upsit_pctl, DATSCAN_PUTAMEN_L, con_striatum, mean_putamen, and DATSCAN_PUTAMEN_R. These features were used consistently across all subsequent ML and LLM experiments.

### Prompt Construction

To evaluate the performance of LLMs, we developed 4 prompting formats that express input variables in plain text (PT), markdown (MD), special token (ST) annotations, and a combined markdown with special token (MD+ST) structure [29]. The PT format presents the input features as natural language sentences, whereas the MD format uses a structured table to delineate characteristics and enhance feature recognition, and the combined MD+ST format integrates both structured layout and explicit ST annotation.

Each prompting format was applied under 0-shot conditions to 3-shot conditions. All few-shot settings incorporated balanced PD and HC examples, with 1 pair for 1-shot, 2 pairs for 2-shot, and 3 pairs for 3-shot prompting. For dual-output prompting, the diagnostic label was followed by 3 sentences of post hoc explanatory text, which served as an exploratory output and did not represent true model interpretability [30].

For dual-output prompting, each LLM was instructed to generate a diagnostic label (PD or HC) followed by 3 sentences of post hoc explanatory text with a maximum length of 180 tokens to ensure comparability across models. Examples of all prompting formats, few-shot configurations, and sample outputs are provided in Multimedia Appendix 3.

### Modeling Approaches

This study compared traditional ML classifiers with multiple LLMs in order to evaluate diagnostic performance under consistent experimental conditions. As deterministic baselines, we trained LR with L2 regularization and a SVM with a radial basis function (RBF) kernel, using the top 10 SHAP-selected features. Both models were implemented using standard scikit-learn procedures without extensive hyperparameter tuning, since the goal was to establish transparent and reproducible baselines rather than to maximize predictive performance. The complete hyperparameter configurations for the ML classifiers are summarized in Multimedia Appendix 4. These models were evaluated following the procedures described in the “Training and Evaluation Settings” section.

For LLM-based classification, we selected model families that collectively capture diversity in architecture, accessibility, and usage constraints. The included families were LLaMA, GPT, Gemini, and Claude. Each family provided one or more model variants with different parameter sizes or intended applications. The LLaMA models consisted of LLaMA 3.1 8B Instruct and LLaMA 3.3 70B Instruct, both of which were run locally using open-access model weights. The GPT models consisted of GPT-4o-mini and GPT-4o, accessed through the OpenAI application programming interface (API). The Gemini models included Gemini 1.5 Flash and Gemini 1.5 Pro, accessed through the Google Gemini API. For the Claude family, the Claude 3.5 Sonnet model was used. Although lightweight variants such as Claude Instant were available, the Sonnet model was selected due to budget considerations and its expected performance.

All LLMs were accessed through their officially supported APIs or locally hosted implementations using Python-based programmatic interfaces. The same prompting formats, shot settings, and evaluation procedures were applied across all models, except where input-format restrictions required specific handling. Additional implementation details, including software environments and API configurations, are provided in Multimedia Appendix 5.

### Training and Evaluation Settings

#### Overview

The evaluation schedule and prompting phases for each model are summarized in Multimedia Appendix 6, and the hardware and software environments used in all experiments are described in Multimedia Appendix 7. To address the class imbalance between PD and HC, model performance was primarily assessed using the *F*_1_-score, with precision and recall reported as supporting metrics. Accuracy was treated as a secondary measure, given that *F*_1_-score provides a more balanced assessment under imbalanced classification tasks [31].

For all binary classification tasks, PD was assigned as the positive class (label=1) and HC as the negative class (label=0) across all ML and LLM experiments. Numerical features were standardized using *z* score scaling, where each variable was transformed by subtracting the mean and dividing by the SD computed from the training split. This ensured a consistent feature scale for both LR and SVM while preventing data leakage. Standardization using *z* score was applied exclusively to the ML baselines. For LLM-based experiments, models were provided with the original clinical values embedded in natural language prompts, rather than normalized numerical vectors. For ML models, performance uncertainty was quantified using 95% bootstrap CIs based on 1000 resampling iterations [32].

For LLMs, diagnostic classification was evaluated under 0-shot settings to 3-shot settings using 4 prompting formats including PT, MD, ST, and MD+ST. All few-shot experiments incorporated balanced PD and HC examples at each shot level. Across all LLM conditions, each participant was evaluated through 30 repeated model executions under identical prompting conditions to account for the inherent nondeterminism of LLM outputs [33]. Any change in diagnostic labels across these executions was recorded as a label inconsistency event. For dual-output prompting experiments, semantic consistency of the generated post hoc explanatory texts was also evaluated.

For each LLM and prompting configuration, macro-averaged performance metrics (*F*_1_-score, precision, recall, and accuracy) were computed for each of the 30 model executions. Nonparametric 95% CIs for LLM performance were estimated only for the temporal validation set using a hierarchical bootstrap procedure. In each iteration, participants were sampled with replacement and, for each selected participant, one prediction was randomly drawn from the 30 repeated trials. The 2.5th and 97.5th percentiles of the resulting performance distributions were reported in the temporal validation tables.

All combinations of prompting format and shot number were evaluated for each LLM on the development test set. The prompting configuration reported as the best for each model corresponds to the combination that achieved the highest macro-averaged *F*_1_-score on this test set. During temporal validation, each LLM was evaluated using only under its best-performing configuration. This ensured that temporal validation assessed the generalizability of each model’s optimal setting rather than retesting all prompt-shot combinations on temporally independent data.

#### ML Baseline Training Protocols

Two conventional ML classifiers were trained as deterministic baselines for comparison with LLM-based diagnostic approaches. The models were LR with L2 regularization and a SVM with an RBF kernel, both of which were trained using the same feature subset identified in the SHAP-based feature selection step. Class weights were applied to address the imbalance between PD and HC samples in the development set.

Both models were implemented using standard scikit-learn procedures without extensive hyperparameters optimization, as the primary goal was to establish transparent and reproducible baselines rather than to maximize predictive performance. LR was trained using the liblinear solver with balanced class weights, while SVM used an RBF kernel with probability estimation enabled. Performance for both models was evaluated for direct comparability with all LLM-based experiments.

#### Few-Shot Prompting Evaluation of LLMs

The LLMs were evaluated using the 4 prompting formats described earlier, applied across 0-, 1-, 2-, and 3-shot settings. Examples of PD and HC were balanced in all few-shot conditions to ensure consistent contextual exposure. All 4 prompting formats were applied to the LLaMA models. In contrast, only the PT and MD formats were applied to the GPT, Gemini, and Claude models because these API-based models do not support ST annotations in their input structure. Importantly, the inclusion of STs substantially influenced both diagnostic performance and output stability. Accordingly, ST-based prompting (including MD+ST) was applied exclusively to the LLaMA models as a model-specific input design choice, and the resulting differences in prompt structuring capability were explicitly considered as a limitation in cross-model comparisons.

#### Dual-Output Prompting of LLMs for Diagnosis and Post Hoc Explanatory Text

This experiment examined whether requiring LLMs to generate post hoc explanatory text could influence their diagnostic classification performance. Recent studies have shown that step-by-step prompting can improve LLM accuracy in complex decision tasks [34], yet fully stepwise reasoning is often impractical in clinical contexts due to token limitations. Rather than instructing the models to articulate their reasoning step by step, the models were prompted to produce concise post hoc explanatory text. This approach enabled assessment of whether a reduced explanatory demand could affect the diagnostic output.

Each model generated a binary diagnosis of PD or HC followed by exactly 3 sentences of post hoc explanatory text. To ensure consistency across models and to manage token usage, the length of these explanatory outputs was fixed. This dual-output prompting was applied to 4 models, including GPT-4o, Claude 3.5 Sonnet, Gemini 1.5 Pro, and LLaMA 3.3 70B. Among these, the LLaMA model was prompted using the ST format, whereas the other models were prompted using the PT format because these models do not support ST inputs.

Although the generated explanations were not reviewed by clinical experts, their semantic consistency was evaluated to assess the stability of the post hoc explanatory text under repeated prompting. High semantic consistency reflects only the reproducibility of the generated text across repeated runs and does not imply that the explanations are clinically accurate or factually grounded. For each participant, their explanatory outputs per model were collected, and pairwise cosine similarity between sentence embeddings was computed using the all-mpnet-base-v2 model from the Sentence-Transformers library [35,36]. This semantic consistency analysis was performed only on the development set, while the temporal validation set was used exclusively for the primary diagnostic evaluation.

#### LLM Supervised Fine-Tuning

Supervised fine-tuning was conducted to evaluate whether labeled training data could improve the diagnostic performance and output stability of lightweight LLMs. Unlike prompt-based learning, which is constrained by the number of examples that can be included within a single prompt, supervised fine-tuning allows the model to learn diagnostic patterns directly from the full training dataset.

Among the models tested in this study, GPT-4o-mini and Gemini 1.5 Flash were selected as compact and computationally efficient alternatives to assess how smaller LLMs perform relative to larger models. Both models were fine-tuned using the same dataset that was used in the prompting experiments, and all evaluations were performed under 0-shot conditions on the identical test set. The generation temperature was fixed at 0.1 to ensure consistent and deterministic behavior. Further details regarding fine-tuning platforms, sampling strategies, and training parameters are provided in Multimedia Appendix 8.

The GPT-4o-mini model was fine-tuned through the OpenAI API. Training jobs were submitted from a local workstation after converting the dataset into JSONL format. A total of 1052 samples were used for training and 186 samples for validation. The fine-tuning process followed OpenAI’s job-based workflow, in which each training job is versioned and automatically made accessible through the OpenAI API upon completion.

The Gemini 1.5 Flash model was fine-tuned using the Google AI Studio platform. Because the platform restricts training to a maximum of 500 samples, stratified sampling was applied to preserve the original class ratio, resulting in 383 PD samples and 117 HC samples. The model was trained for 1 epoch with a batch size of 1 and a learning rate of 1, after which the fine-tuned model was deployed via the Gemini API using the assigned model identifier.

All fine-tuned models were trained and evaluated using the PT prompt format to maintain consistency between training and testing conditions.

### Ethical Considerations

This study used publicly available, deidentified data provided by PPMI. According to institutional policies and the PPMI Data Use Agreement, additional institutional review board approval was not required for secondary analyses of this publicly accessible dataset. The original PPMI study was conducted under institutional review board approval at all participating institutions, and all participants provided written informed consent permitting data sharing and secondary analyses. All analyses in this study were conducted on anonymized records with no personally identifiable information, and no participant compensation was applicable.

## Results

### ML Baseline Performance

To establish a deterministic baseline for comparison with LLM-based diagnostic approaches, we evaluated 2 conventional ML classifiers trained on the top 10 SHAP-selected features. LR and a SVM with an RBF kernel were implemented using standard scikit-learn procedures.

As summarized in Table 3, both models achieved identical performance on the test subset of the development set, yielding a macro-averaged *F*_1_-score of 0.960 and an accuracy of 0.975. Precision and recall were balanced across PD and HC, and the 95% CIs ranged from approximately 0.94 to 1.00, reflecting the modest sample size of the test subset (n=122).

**Table 3:** Performance of machine learning models on the test subset of the development set (n=122; CIs were estimated via 1000 bootstrap iterations).

Model	*F*_1_-score^a^ (95% CI)	Precision (macro avg^b^/PD^c^/HC^d^)	Recall (macro avg/PD/HC)	Accuracy
LR_L2^e^	0.960 (0.943-1.000)	0.953/0.990/0.917	0.968/0.980/0.957	0.975
SVM_RBF^f^	0.960 (0.944-1.000)	0.953/0.990/0.917	0.968/0.980/0.957	0.975

^a^*F*_1_-scores represent macro avg values across PD and HC classes.

^b^macro avg: macro-averaged.

^c^PD: Parkinson disease.

^d^HC: healthy controls.

^e^LR_L2: logistic regression (L2 regularization).

^f^SVM_RBF: support vector machine (RBF kernel).

Table 4 presents the results from the temporal validation set (n=31). LR demonstrated moderately high generalizability, achieving a macro-averaged *F*_1_-score of 0.903 and an accuracy of 0.903. In contrast, SVM showed a substantial decline in generalizability, producing a macro-averaged *F*_1_-score of 0.484 with markedly reduced recall for PD. The wide CIs observed for both models reflect the limited size of the temporal validation set and the inherent uncertainty associated with evaluating performance on small external samples.

**Table 4:** Performance of machine learning models on the temporal validation set (n=31; CIs were estimated via 1000 bootstrap iterations).

Model	*F*1-score^a^ (95% CI)	Precision (macro avg^b^/PD^c^/HC^d^)	Recall (macro avg/PD/HC)	Accuracy
LR_L2^e^	0.903 (0.791-1.000)	0.917/0.833/1.0	0.906/1.0/0.813	0.903
SVM_RBF^f^	0.484 (0.157-0.506)	0.241/0.483/0	0.5/0/1.0	0.484

^a^*F*_1_-scores represent macro avg values across PD and HC classes.

^b^macro avg: macro-averaged.

^c^PD: Parkinson disease.

^d^HC: healthy controls.

^e^LR_L2: logistic regression (L2 regularization).

^f^SVM_RBF: support vector machine (RBF kernel).

Given the small size of the temporal validation set (n=31), results obtained on this dataset should be interpreted with caution, as performance estimates may be sensitive to individual misclassifications.

**Figure 2:**
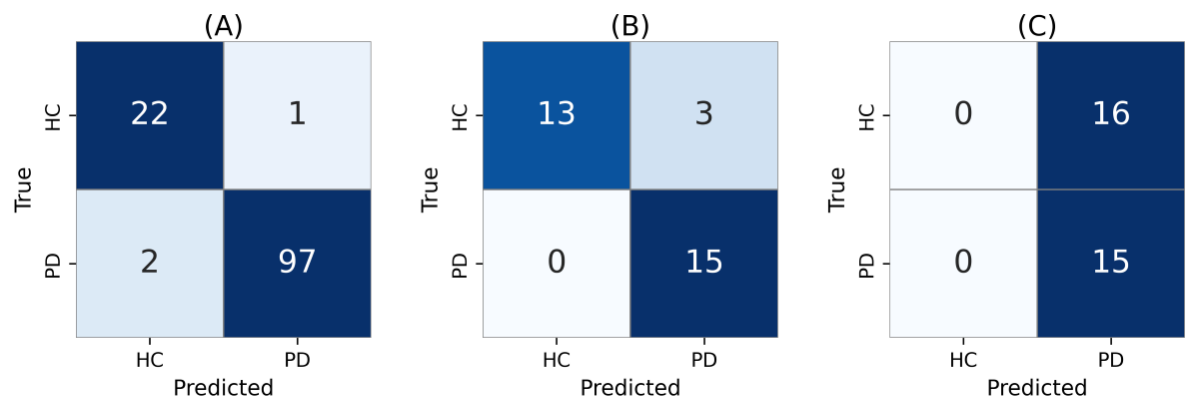
Confusion matrix for machine learning classifiers. (A) Test subset of the development set. (B) Logistic regression of temporal validation set. (C) Support vector machine of temporal validation set. HC: healthy controls; PD: Parkinson disease.

Figure 2 illustrates the prediction patterns for the 2 classifiers across both datasets. On the development test subset (Figure 2A), both models produced identical predictions, misclassifying 1 HC participant as PD and misclassifying 2 PD participants as HC. In the temporal validation set, LR misclassified only 3 HC cases as PD (Figure 2B), whereas SVM misclassified nearly all HC cases (Figure 2C). Although the two models performed similarly on the development test subset, their stability diverged substantially when evaluated on data collected at a later timepoint.

### Few-Shot Prompting Performance of LLMs

A total of 4 prompting formats, including PT, MD, ST, and MD+ST, were evaluated under 0-shot conditions to 3-shot conditions to assess the diagnostic performance of LLMs. ST-based prompts were applied exclusively to the LLaMA models because the inclusion of STs substantially affected both classification accuracy and output stability. Non-LLaMA models such as GPT, Gemini, and Claude did not support ST formatting and were therefore evaluated using PT and MD formats.

Table 5 presents the best-performing results for each model on the test dataset (n=122). The best-performing configuration for each model corresponds to the prompt type and shot number that achieved the highest macro-averaged *F*_1_-score on the development test set.

LLaMA 3.3 70B and Gemini 1.5 Pro achieved the highest macro-averaged *F*_1_-score of 0.987 with an accuracy of 0.992 and stable predictions across 30 repeated trials. LLaMA 3.1 8B reached the same *F*_1_-score using the MD+ST prompt at the 3-shot setting but showed one instance of prediction inconsistency. Claude 3.5 Sonnet demonstrated strong performance with an *F*_1_-score of 0.972 and an accuracy of 0.984 under 1-shot MD setting, maintaining complete consistency across repeated runs. GPT-4o and GPT-4o-mini achieved slightly lower but stable performance, with macro-averaged *F*_1_-scores of 0.961 and 0.910, respectively. Gemini 1.5 Flash produced the lowest *F*_1_-score (0.894) despite consistent predictions, reflecting reduced sensitivity for HC cases. Detailed per-participant consistency analyses across 30 trials are provided in Multimedia Appendix 9.

**Figure 3:**
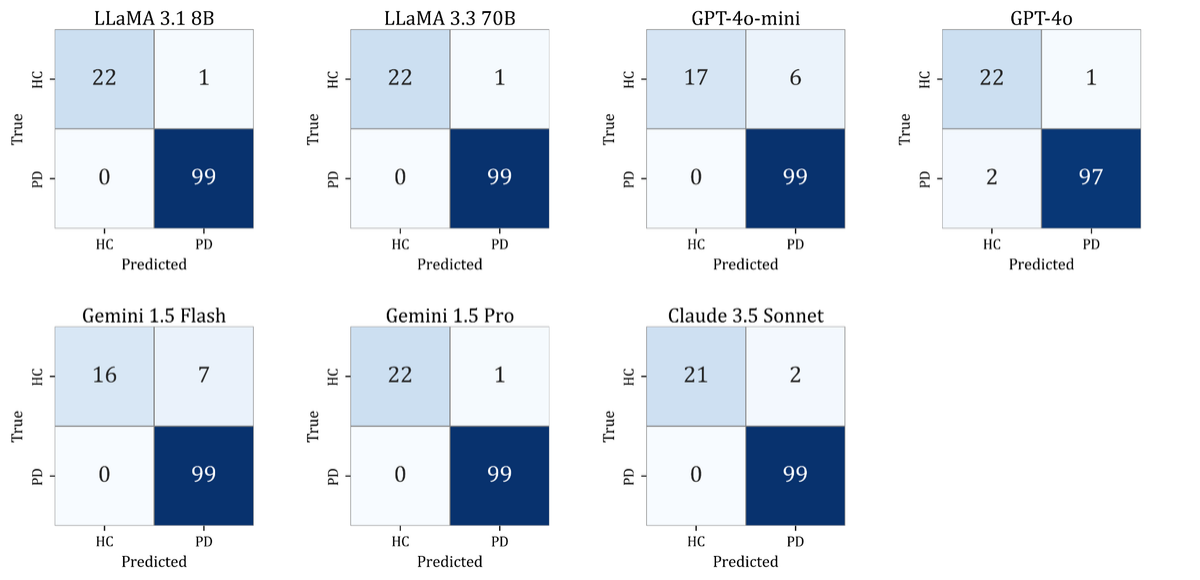
Confusion matrices of large language models under the best-performing configurations on the test dataset (n=122). HC: healthy controls; PD: Parkinson disease.

Figure 3 displays the confusion matrices for the best-performing configuration of each model. Most classification errors occurred in HC samples, indicating high sensitivity for PD across the LLMs.

**Table 5:** Best performance of large language models on the test dataset (n=122).

Model	Prompt	Shot	*F*_1_-score^a^	Precision (macro avg^b^/PD^c^/HC^d^)	Recall (macro avg/PD/HC)	Accuracy	Inconsistency^e^, n	Inconsistent cases^f^
LLaMA 3.1 8B	MD+ST^g^	3	0.987	0.995/0.990/1	0.978/1/0.957	0.992	1	PD #73 (13:17)
LLaMA 3.3 70B	ST^h^	2	0.987	0.995/0.990/1	0.978/1/0.957	0.992	0	—^i^
GPT-4o-mini	MD^j^	2	0.910	0.972/0.943/1	0.869/1/0.739	0.951	1	HC #103 (19:11)
GPT-4o	PT^k^	0	0.961	0.954/0.990/0.917	0.969/0.980/0.957	0.975	2	HC #26 (28:2); HC #68 (17:13)
Gemini 1.5 Flash	PT, MD	2	0.894	0.967/0.934/1	0.848/1/0.696	0.938	0	—
Gemini 1.5 Pro	PT, MD	2	0.987	0.995/0.990/1	0.978/1/0.957	0.992	0	—
Claude 3.5 Sonnet	MD	1	0.972	0.990/0.980/1	0.957/1/0.913	0.984	0	—

^a^*F*_1_-scores represent macro avg values across PD and HC classes.

^b^macro avg: macro-averaged.

^c^PD: Parkinson disease.

^d^HC: healthy controls.

^e^Number of participants (out of 122) whose predictions were inconsistent at least once across 30 repeated trials.

^f^Example of inconsistent participants showing the final label (eg, PD #73) and the number of predicted labels across 30 runs (eg, 13:17 indicates 13 HC and 17 PD predictions).

^g^MD+ST: markdown with special token.

^h^ST: special token.

^i^Not applicable.

^j^MD: markdown.

^k^PT: plain text.

**Figure 4:**
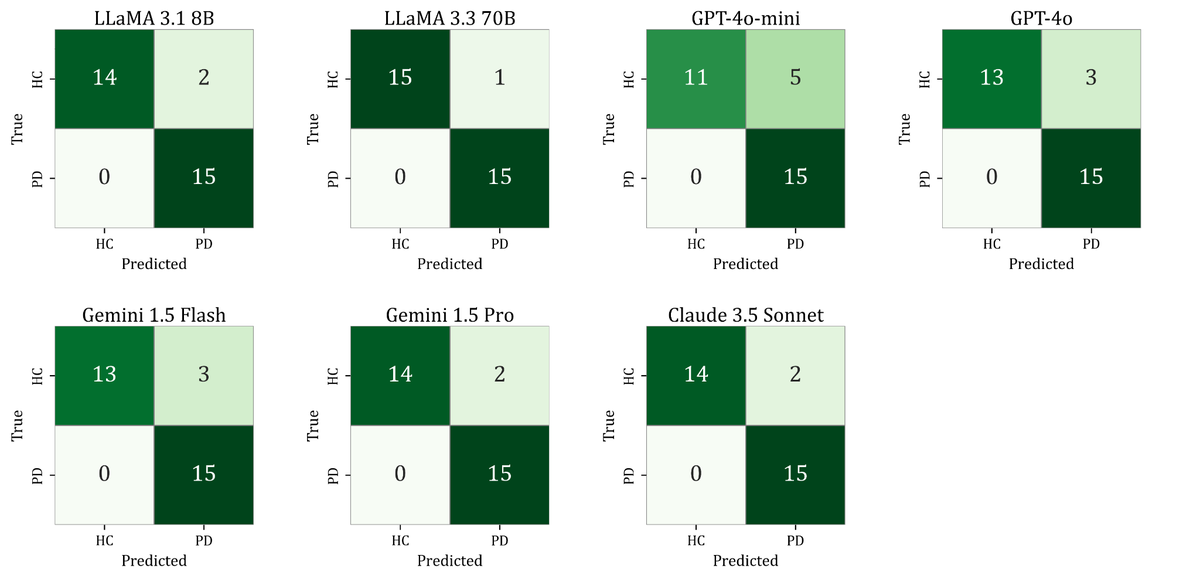
Confusion matrices of large language models under their best-performing configurations on the temporal validation set (n=31). HC: healthy controls; PD: Parkinson disease.

Table 6 and Figure 4 summarize the best-performing configuration of each LLM on the temporal validation set (n=31). LLaMA 3.3 70B achieved the highest macro-averaged *F*_1_-score of 0.968 using the ST format at the 2-shot setting, with recall of 1.00 and consistent predictions across all participants. LLaMA 3.1 8B, Gemini 1.5 Pro, and Claude 3.5 Sonnet showed comparable performance, each achieving a macro-averaged *F*_1_-score of 0.936 with stable predictions. GPT-4o and Gemini 1.5 Flash achieved moderately high performance (*F*_1_-score=0.903), with consistent predictions across all participants. In contrast, GPT-4o-mini achieved the lowest *F*_1_-score (0.836), driven primarily by reduced recall for HC cases. Across all models, recall for PD remained consistently high (recall≥0.938), demonstrating strong sensitivity even when evaluated on data collected at a later timepoint. Figure 4 shows the confusion matrices for each model under the prompt and shot condition listed in Table 6.

These findings indicate that LLMs generally maintained high diagnostic accuracy and prediction stability under temporally separated data, supporting their potential generalizability beyond the development dataset.

**Table 6:** Best performance of large language models on the temporal validation set (n=31).

Model	Prompt	Shot	*F*_1_-score^a^ (95% CI)	Precision (macro avg^b^/PD^c^/HC^d^)	Recall (macro avg/PD/HC)	Accuracy	Inconsistency^e^, n	Inconsistent cases^f^
LLaMA 3.1 8B	MD+ST^g^	3	0.936 (0.854-1.000)	0.941/0.882/1	0.938/1/0.875	0.935	2	HC #13 (9:21); HC #23 (7:23)
LLaMA 3.3 70B	ST^h^	2	0.968 (0.899-1.000)	0.969/0.938/1	0.969/1/0.938	0.968	0	—^i^
GPT-4o-mini	MD^j^	2	0.836 (0.708-0.966)	0.875/0.750/1	0.844/1/0.688	0.839	2	HC #10 (4:26); HC #28 (8:22)
GPT-4o	PT^k^	0	0.903 (0.773-1.000)	0.917/0.833/1	0.906/1/0.813	0.903	2	PD #1 (1:29); HC #10 (28:2)
Gemini 1.5 Flash	PT	2	0.903 (0.773-1.000)	0.917/0.833/1	0.906/1/0.813	0.903	0	—
Gemini 1.5 Pro	PT	2	0.936 (0.832-1.000)	0.941/0.882/1	0.938/1/0.875	0.935	0	—
Claude 3.5 Sonnet	MD	1	0.936 (0.832-1.000)	0.941/0.882/1	0.938/1/0.875	0.935	31	—

^a^*F*_1_-scores represent macro avg values across PD and HC classes.

^b^macro avg: macro-averaged.

^c^PD: Parkinson disease.

^d^HC: healthy controls.

^e^Number of participants (out of 31) whose predictions were inconsistent at least once across 30 repeated trials.

^f^Example of inconsistent participants showing the final label (eg, HC #13) and the number of predicted labels across 30 runs (eg, 9:21 indicates 9 HC and 21 PD predictions).

^g^MD+ST: markdown with special token.

^h^ST: special token.

^i^Not applicable.

^j^MD: markdown.

^k^PT: plain text.

### Diagnostic Performance Under Dual-Output Prompting

Dual-output prompting was used to evaluate whether requiring LLMs to generate post hoc explanatory text influenced their diagnostic reliability. This experimental setting included 4 representative models, namely LLaMA 3.3 70B, GPT-4o, Gemini 1.5 Pro, and Claude 3.5 Sonnet. Unlike diagnostic-only prompting, this setup instructed each model to output a binary diagnostic label followed by 3 sentences of post hoc explanatory text. Because the LLaMA models are highly sensitive to input formatting, dual-output prompts for LLaMA 3.3 70B were constructed using the ST format. GPT-4o, Gemini 1.5 Pro, and Claude 3.5 Sonnet do not support ST inputs, and therefore PT prompts were used for these models to maintain compatibility.

As summarized in Table 7, all models achieved high diagnostic performance under dual-output prompting, although overall *F*_1_-scores were slightly lower than those obtained under few-shot prompting. LLaMA 3.3 70B and Claude 3.5 Sonnet each achieved a macro-averaged *F*_1_-score of 0.972 and correctly identified all PD cases. GPT-4o and Gemini 1.5 Pro showed similar results and achieved *F*_1_-scores of 0.958 with an accuracy of 0.975. Most prediction errors occurred in HC cases, whereas all PD samples were correctly classified. Instances of inconsistency across 30 repeated trials were rare, typically affecting no more than 2 participants for each model. The full repeated-trial results are presented in Multimedia Appendix 10.

**Figure 5:**
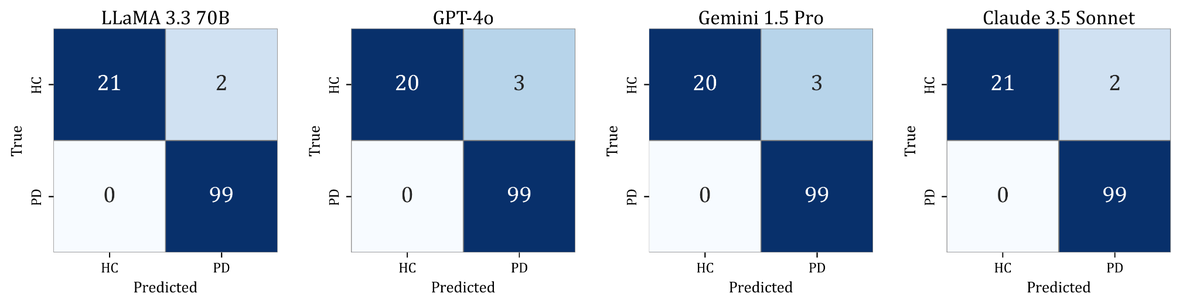
Confusion matrices of large language models under best-performing dual-output prompting conditions on the test subset of the development set (n=122). HC: healthy controls; PD: Parkinson disease.

Figure 5 displays the confusion matrices corresponding to each model’s best-performing dual-output configuration.

**Table 7:** Best performance of large language models on the test dataset under dual-output prompting (n=122).

Model	Prompt	Shot	*F*_1_-score^a^	Precision (macro avg^b^/PD^c^/HC^d^)	Recall (macro avg/PD/HC)	Accuracy	Inconsistency^e^, n	Inconsistent cases^f^
LLaMA 3.3 70B	ST^g^	3	0.972	0.990/0.980/1	0.957/1/0.913	0.984	122	HC #23 (29:1); HC #68 (28:2)
GPT-4o	PT^h^	1	0.958	0.985/0.970/1	0.935/1/0.870	0.975	122	HC #26 (26:4); HC #77 (6:24)
Gemini 1.5 Pro	PT	3	0.958	0.985/0.970/1	0.935/1/0.870	0.975	122	HC #97 (28:2)
Claude 3.5 Sonnet	PT	0	0.972	0.990/0.980/1	0.957/1/0.913	0.984	122	PD #23 (1:29)

^a^*F*_1_-scores represent macro avg values across PD and HC classes.

^b^macro avg: macro-averaged.

^c^PD: Parkinson disease.

^d^HC: healthy controls.

^e^Number of participants (out of 122) whose predictions were inconsistent at least once across 30 repeated trials.

^f^Example of inconsistent participants showing the final label (eg, HC #23) and the number of predicted labels across 30 runs (eg, 29:1 indicates 29 HC and 1 PD predictions).

^g^ST: special token.

^h^PT: plain text.

Generalizability was further assessed using the temporal validation set (n=31). As shown in Table 8, all models maintained strong diagnostic sensitivity, and recall for PD remained equal to 1 across all participants. GPT-4o achieved the highest macro-averaged *F*_1_-score of 0.968 with an accuracy of 0.968. LLaMA 3.3 70B followed with a macro-averaged *F*_1_-score of 0.935. Gemini 1.5 Pro and Claude 3.5 Sonnet showed modest decreases in performance with *F*_1_-scores of 0.903 and 0.869, respectively, primarily due to HC misclassifications.

**Figure 6:**
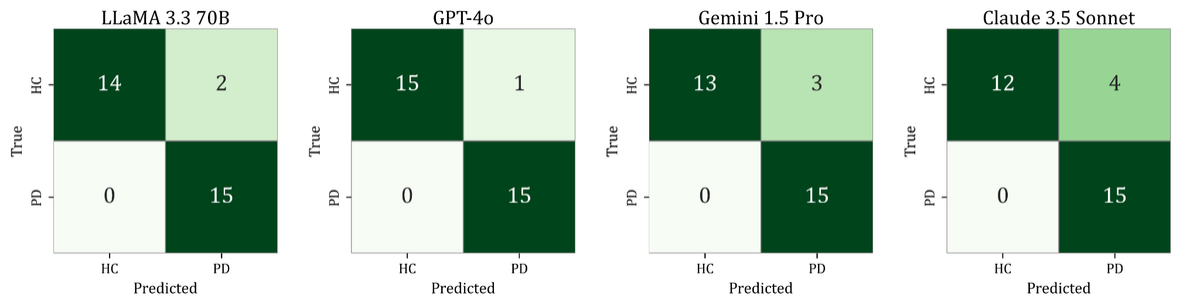
Confusion matrices of large language models under best-performing dual-output prompting conditions on the temporal validation set (n=31). HC: healthy controls; PD: Parkinson disease.

Figure 6 presents the confusion matrices for the temporal validation set under the best-performing configuration for each model.

**Table 8:** Best performance of large language models under dual-output prompting on the temporal validation set (n=31).

Model	Prompt	Shot	*F*_1_-score^a^ (95% CI)	Precision (macro avg^b^/PD^c^/HC^d^)	Recall (macro avg/PD/HC)	Accuracy	Inconsistency^e^	Inconsistent cases^f^
LLaMA 3.3 70B	ST^g^	3	0.935 (0.832-1.000)	0.941/0.882/1	0.938/1/0.875	0.935	2	HC #11 (23:7); HC #21 (8:22)
GPT-4o	PT^h^	1	0.968 (0.896-1.000)	0.969/0.938/1	0.969/1/0.938	0.968	1	HC #21 (29:1)
Gemini 1.5 Pro	PT	3	0.903 (0.774-1.000)	0.917/0.833/1	0.906/1/0.813	0.903	0	—^i^
Claude 3.5 Sonnet	PT	0	0.869 (0.735-0.968)	0.895/0.789/1	0.875/1/0.750	0.871	1	HC #10 (1:29)

^a^*F*_1_-scores represent macro avg values across PD and HC classes.

^b^macro avg: macro-averaged.

^c^PD: Parkinson disease.

^d^HC: healthy controls.

^e^Number of participants (out of 31) whose predictions were inconsistent at least once across 30 runs.

^f^Example of inconsistent participants showing the final label (eg, HC #11) and the number of predicted labels across 30 runs (eg, 23:7 indicates 23 HC and 7 PD predictions).

^g^ST: special token.

^h^PT: plain text.

^i^Not applicable.

Following the diagnostic classification results presented in Table 7, the semantic consistency of the post hoc explanatory texts was evaluated to assess the stability of generated explanations under dual-output prompting. Pairwise cosine similarity was computed among 30 post hoc explanatory texts generated for each participant, and the resulting mean and SD were averaged across all 122 test participants. As shown in Table 9, all models maintained high semantic consistency, with mean cosine similarity values exceeding 0.95. LLaMA 3.3 70B achieved the highest value under 0-shot prompting (0.997 ± 0.005), and the variation across models and shot settings was small (≤ 0.03). This evaluation was limited to the development set, and additional exploratory metrics for the temporal validation set are provided in Multimedia Appendix 11.

**Table 9:** Semantic consistency of reasoning outputs on the test dataset (n=122). Semantic consistency was assessed based on pairwise cosine similarity among 30 post hoc explanatory texts generated per participant.

Model		Cosine similarity, mean (SD)
**LLaMA 3.3 70B**		
	0-shot	0.997 (0.005)
	1-shot	0.995 (0.009)
	2-shot	0.966 (0.016)
	3-shot	0.980 (0.011)
**GPT-4o**		
	0-shot	0.973 (0.016)
	1-shot	0.980 (0.011)
	2-shot	0.981 (0.011)
	3-shot	0.975 (0.016)
**Gemini 1.5 Pro**		
	0-shot	0.969 (0.019)
	1-shot	0.968 (0.025)
	2-shot	0.956 (0.029)
	3-shot	0.951 (0.026)
**Claude 3.5 Sonnet**		
	0-shot	0.985 (0.017)
	1-shot	0.986 (0.016)
	2-shot	0.987 (0.016)
	3-shot	0.979 (0.015)

### Fine-Tuned Prompting Performance of LLMs

Supervised fine-tuning substantially improved the diagnostic performance of lightweight LLMs and resulted in more consistent classification across the evaluated datasets. Table 10 summarizes the results of GPT-4o-mini and Gemini 1.5 Flash on the development test set (n=122).

**Table 10:** Fine-tuning performance of lightweight large language models on the development test set (n=122).

Model	Prompt	*F*_1_-score^a^	Precision (macro avg^b^/PD^c^/HC^d^)	Recall (macro avg/PD/HC)	Accuracy	Inconsistency^e^	Inconsistent cases^f^
GPT-4o-mini	PT^g^	0.987	0.995 / 0.990 / 1	0.978 / 1 / 0.957	0.992	0	—^h^
Gemini 1.5 Flash	PT	0.973	0.990 / 0.980 / 1	0.957 / 1 / 0.913	0.984	1	HC #23 (9:21)

^a^*F*_1_-scores represent macro avg values across PD and HC classes.

^b^macro avg: macro-averaged.

^c^PD: Parkinson disease.

^d^HC: healthy controls.

^e^Number of participants (out of 122) whose predictions were inconsistent at least once across 30 repeated trials.

^f^Example of inconsistent participants showing the final label (eg, HC #23) and the number of predicted labels across 30 runs (eg, 9:21 indicates 9 HC and 21 PD predictions).

^g^PT: plain text.

^h^Not applicable.

**Figure 7:**
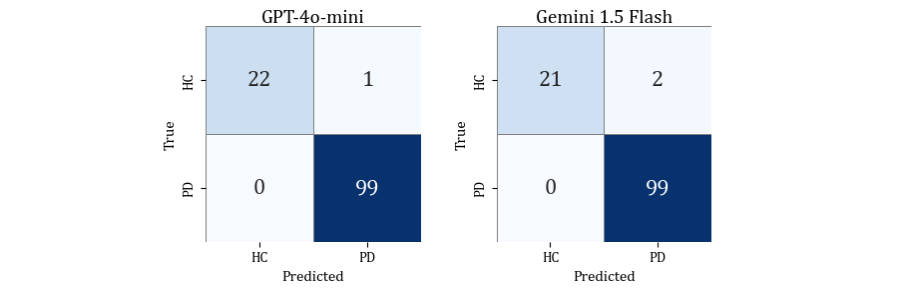
Confusion matrices of lightweight large language models after fine-tuning on the development test set (n=122). HC: healthy controls; PD: Parkinson disease.

The fine-tuned GPT-4o-mini achieved the highest macro-averaged *F*_1_-score of 0.987, with recall of 1.00 and stable predictions across 30 repeated trials. Gemini 1.5 Flash followed closely with an *F*_1_-score of 0.973, maintaining an accuracy of 0.984 and showing only 1 inconsistent prediction (participant HC #23). Both models were trained and evaluated using PT prompts. The corresponding confusion matrices are shown in Figure 7. Figure 7 displays the confusion matrices of the fine-tuned LLMs on the development test set, showing that nearly all misclassifications occurred among HC participants, whereas both models correctly identified all PD cases in the sample.

**Figure 8:**
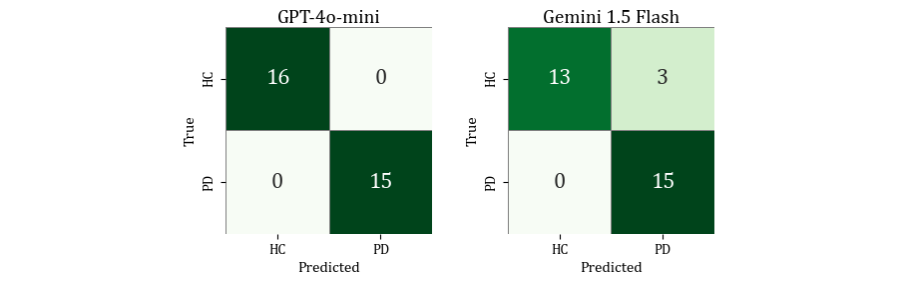
Confusion matrices of lightweight large language models after fine-tuning on the temporal validation set (n=31). HC: healthy controls; PD: Parkinson disease.

To further assess model generalizability, both fine-tuned LLMs were evaluated on the temporal validation set (n=31). As summarized in Table 11, GPT-4o-mini achieved *F*_1_-score, precision, recall, and accuracy of 1.000 on the temporal validation set, correctly classifying all participants in this sample. In contrast, Gemini 1.5 Flash demonstrated slightly lower but still strong performance with an *F*_1_-score of 0.903 and an accuracy of 0.903. All PD participants were correctly identified, and the few errors were limited to HC participants. Figure 8 presents the confusion matrices under the same fine-tuned PT prompting configuration. These findings confirm that fine-tuning notably improved classification stability while preserving sensitivity to PD in temporally independent data.

**Table 11:** Fine-tuning performance of lightweight large language models on the temporal validation set (n=31).

Model	Prompt	*F*_1_-score^a^ (95% CI)	Precision (macro avg^b^/PD^c^/HC^d^)	Recall (macro avg/PD/HC)	Accuracy	Inconsistency^e^	Inconsistent cases^f^
GPT-4o-mini	PT^g^	1 (1.000-1.000)	1/1/1	1/1/1	1	0	—^h^
Gemini 1.5 Flash	PT	0.903 (0.770-1.000)	0.917/0.833/1	0.906/1/0.813	0.903	1	HC #21 (25:5)

^a^*F*_1_-scores represent macro avg values across PD and HC classes.

^b^macro avg: macro-averaged.

^c^PD: Parkinson disease.

^d^HC: healthy controls.

^e^Number of participants (out of 31) whose predictions were inconsistent at least once across 30 runs.

^f^Example of inconsistent participants showing the final label (eg, HC #21) and the number of predicted labels across 30 runs (eg, 25:5 indicates 25 HC and 5 PD predictions).

^g^PT: plain text.

^h^Not applicable.

These results confirm that with sufficient training data and consistent prompting formats, even small-scale LLMs can achieve classification accuracy comparable to larger models while maintaining stable performance.

**Figure 9:**
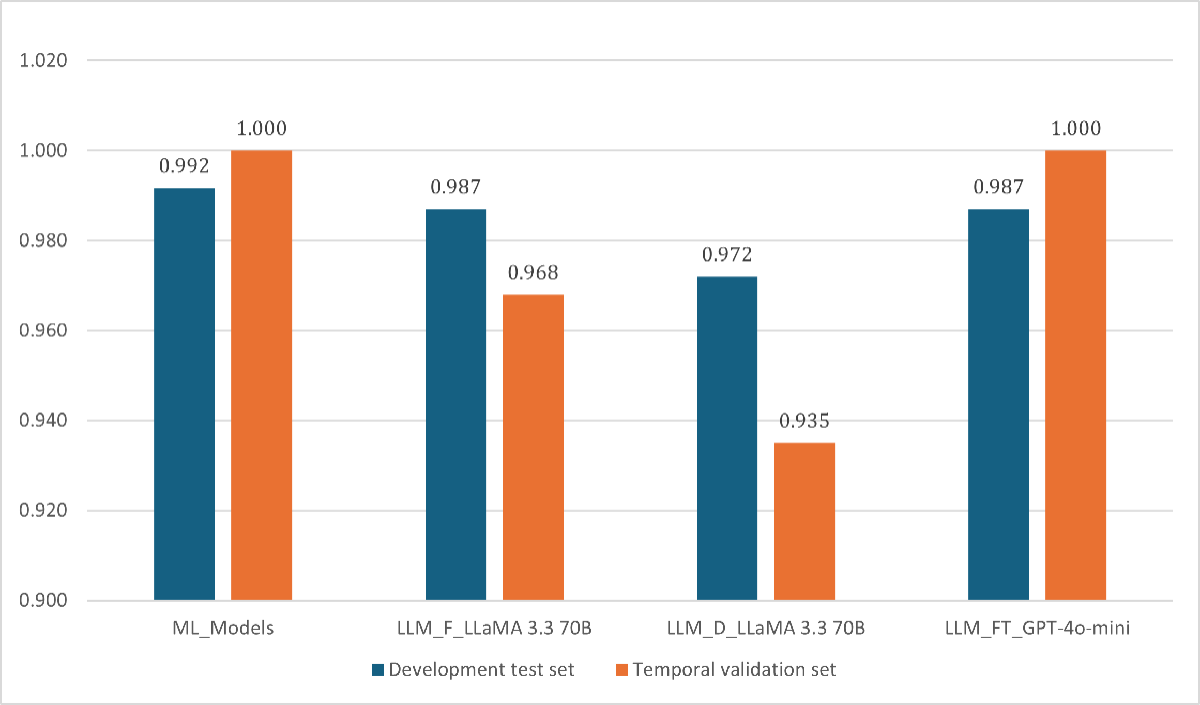
Comparison of the best macro-averaged F1-scores from the top-performing models in machine learning (ML) and large language model experiments across few-shot, dual-output, and fine-tuned prompting on the development and temporal validation sets.

Figure 9 provides an integrated comparison of the best-performing models across all experimental settings, including traditional ML baselines, few-shot prompting with LLM (LLM_F), dual-output prompting with LLM (LLM-D), and fine-tuned prompting with LLM (LLM_FT). Across the development dataset, multiple LLM configurations achieved macro-averaged *F*_1_-scores comparable to LR, and fine-tuned lightweight models reached the highest overall performance.

On the temporal validation dataset, LR maintained moderate generalizability, whereas SVM showed substantial degradation when applied to temporally separated data. In contrast, several LLM configurations preserved high recall for PD and sustained overall performance, particularly under fine-tuned and few-shot prompting conditions. These results demonstrate that while ML baselines provide deterministic reference points, LLMs exhibit greater flexibility across prompting strategies and maintain stable sensitivity to PD in both datasets.

## Discussion

### Overview

This study examined how modern LLMs process structured clinical variables when these variables are reformatted into natural language prompts for the diagnostic classification of PD. Using SHAP-selected features derived from the PPMI dataset, we compared multiple LLM families and prompting strategies with conventional ML baselines. Three main findings emerged. First, several LLMs achieved diagnostic performance comparable to LR while maintaining high sensitivity for PD across both the development test set and the temporal validation set. Second, the diagnostic behavior of LLMs varied depending on prompt format, model family, and shot configuration, whereas the ML baselines produced deterministic and highly stable predictions. Third, supervised fine-tuning markedly improved both accuracy and output stability in lightweight LLMs, allowing a compact model such as GPT-4o-mini to correctly classify all participants in the temporal validation set. In addition, because the ML baselines in this study were minimally tuned, part of any performance gap between ML models and LLMs may reflect limited optimization of the ML baselines rather than true methodological differences. Accordingly, the present comparisons should be interpreted as exploratory rather than definitive.

The performance of the LLaMA family was strongly influenced by input formatting. In particular, the inclusion or removal of STs resulted in notable differences in accuracy and sensitivity, although their classification results varied more widely across different shot settings [29]. Dual-output prompting, which required models to generate diagnostic labels along with post hoc explanatory text, resulted in slightly lower *F*_1_-scores compared with diagnostic-only prompting but did not substantially destabilize predictions. The generated text exhibited high semantic consistency across repeated trials. These explanations should be regarded as post hoc natural language outputs rather than indicators of true model interpretability, since they are produced after the model’s primary diagnostic prediction step [14].

To evaluate whether inconsistent predictions reflected clinically ambiguous participants rather than model-level variability, we conducted a qualitative review of cases that exhibited the highest numbers of label inconsistencies. A total of 2 HC participants and 2 PD participants were selected for detailed examination. All 10 SHAP-selected variables, including Unified Parkinson Disease Rating Scale (UPDRS) motor scores, University of Pennsylvania Smell Identification Test (UPSIT) percentiles, and dopamine transporter single-photon emission computed tomography putaminal uptake metrics, were compared with the overall distributions of the PD and HC groups. None of the reviewed cases demonstrated borderline or contradictory clinical profiles. The HC cases showed preserved dopaminergic uptake and normal motor assessments, with only mild olfactory reductions typical of healthy older adults. The PD cases exhibited reduced dopaminergic activity, clear asymmetry, and motor impairment consistent with established PD patterns. These observations suggest that label inconsistencies are unlikely to arise from underlying clinical ambiguity. Instead, they appear to reflect model stochasticity and prompt-dependent variability [33].

Supervised fine-tuning clarified the role of training data in stabilizing LLM predictions. When provided with labeled examples, both GPT-4o-mini and Gemini 1.5 Flash demonstrated substantial improvements in diagnostic accuracy and showed consistently high sensitivity to PD on the temporal validation set. GPT-4o-mini classified all 31 participants correctly after fine-tuning. This result suggests that compact models can approximate or exceed the performance of larger models when trained on appropriately structured datasets. It also indicates that fine-tuning can reduce susceptibility to prompt-level variability and may support more reliable behavior in clinical decision-support environments. However, this comparison should be interpreted with caution. Although architectural differences may also contribute to the observed performance gap, GPT-4o-mini was fine-tuned on more than twice as many labeled samples as Gemini 1.5 Flash (1052 vs 500) due to platform constraints. Part of GPT-4o-mini’s superior performance may therefore reflect the larger amount of training data rather than inherent model advantages, which limits how directly the two models can be compared.

Overall, this study illustrates both the potential and the limitations of modern LLMs for processing structured clinical variables that are presented in natural language form. While several models achieved strong diagnostic performance and generalized well to temporally separated data, their outputs remained sensitive to prompt structures, model architectures, and few-shot configurations. Occasional inconsistencies across repeated runs further highlight the stochastic nature of LLM output generation [33]. These characteristics reinforce the importance of careful interpretation and the need for rigorous evaluation frameworks before LLMs can be integrated safely into real-world diagnostic workflows.

### Limitations

Several limitations should be considered when interpreting these findings. First, although temporal validation provided an important assessment of model generalizability, the temporal validation set was relatively small (n=31), which resulted in wide CIs for the reported performance metrics. In addition, models were trained on datasets that included imputed values, whereas evaluation was conducted on datasets restricted to complete cases without missing data, including both the development test set and the temporal validation set. This mismatch may introduce a distributional shift and result in performance estimates that reflect a best-case evaluation scenario rather than real-world clinical conditions where missing data are common.

Second, the 10 features used for model input were selected using SHAP values from tree-based models. Feature sets obtained using alternative selection strategies may differ, so the current feature subset may not fully represent model-agnostic feature selection. Furthermore, the explanatory text generated by LLMs under the dual-output prompting framework was not reviewed by clinical experts. Accordingly, the semantic consistency metric reflects internal textual stability rather than clinically accurate or factually grounded post hoc explanatory text, and the clinical validity of the generated explanations remains unverified.

Finally, several methodological constraints limit direct model-to-model comparisons. Prompt format and shot configuration were selected based on performance observed on the development test dataset, rather than using a separate validation set for configuration selection. This design choice reflects the study’s aim to broadly compare model behaviors rather than to establish definitive optimal configurations. In addition, platform-specific constraints limited the extent of fine-tuning that could be performed across LLMs. While GPT-4o-mini was fine-tuned using 1052 training samples with an additional held-out validation set of 186 samples, the Gemini 1.5 Flash fine-tuning interface restricts supervised training to a maximum of 500 samples, and the machine learning baselines were trained on 990 samples. As a result, the observed performance differences across models may reflect differences in training data availability rather than inherent architectural superiority and should be interpreted as exploratory.

Prompt structuring flexibility also differed across platforms, as the ST format was applied only to the LLaMA models due to platform-specific input constraints, further limiting the degree to which direct model-to-model comparisons can be made in this study.

### Future Work

Future research should expand these results in several directions. Larger temporal or external datasets, including real-world clinical settings, are needed to strengthen generalizability assessments. Additional work is warranted to examine optimization strategies for prompt design, temperature settings, and calibration methods that may reduce stochastic variability. Expert-based evaluation of generated post hoc explanatory text may clarify how these outputs can be used to support clinical decision-making. Further exploration of supervised fine-tuning for additional lightweight LLMs could help identify resource-efficient models suitable for deployment in constrained clinical environments. Finally, integrating imaging, sensor-derived digital biomarkers, and longitudinal clinical trajectories may clarify how LLMs can combine multimodal biomedical data for diagnostic tasks.

### Conclusions

This study provides an exploratory benchmark of how LLMs process structured clinical variables when presented in natural language form. Multiple LLMs achieved diagnostic performance comparable to conventional ML baselines and maintained high sensitivity for PD under temporal validation. However, their predictions were influenced by prompt format, shot configuration, and model architecture, and occasional inconsistencies reflected inherent stochasticity rather than clinical ambiguity. Supervised fine-tuning substantially improved reliability in lightweight models, demonstrating that compact architectures can achieve stable and high-performing classification when trained on sufficient labeled examples. These findings highlight both the opportunities and the challenges associated with applying LLMs to structured clinical data and emphasize the need for rigorous evaluation before clinical implementation.

## Appendix

Multimedia Appendix 1 Performance and weights of tree-based models.

Multimedia Appendix 2 Weighted mean SHAP values for the top 10 selected variables.

Multimedia Appendix 3 Prompt formats by structure type and output design.

Multimedia Appendix 4 Hyperparameter configurations for logistic regression and support vector machine classifiers.

Multimedia Appendix 5 Description of model families and API.

Multimedia Appendix 6 Experimental timeline of model evaluations.

Multimedia Appendix 7 Hardware and software configuration.

Multimedia Appendix 8 Summary of fine-tuning configurations for lightweight language models.

Multimedia Appendix 9 Diagnostic performance of large language models in Parkinson disease classification using prompt engineering and few-shot learning.

Multimedia Appendix 10 Diagnostic generalization of large language models under dual-output prompting on the test dataset.

Multimedia Appendix 11 Semantic consistency of reasoning outputs on the temporal validation set under dual-output prompting.

Multimedia Appendix 12 Complete code and dataset package used for all experiments, including preprocessing, few-shot prompting, reasoning consistency analysis, and supervised fine-tuning.

## Abbreviations

API: application programming interface
HC: healthy controls
LLM: large language model
LLM_D: dual-output prompting with LLM
LLM_F: few-shot prompting with LLM
LLM_FT: fine-tuned prompting with LLM
LR: logistic regression
MD: markdown
MD+ST: markdown with special token
ML: machine learning
PD: Parkinson disease
PPMI: Parkinson’s Progression Markers Initiative
PT: plain text
RBF: radial basis function
SHAP: Shapley additive explanations
ST: special token
SVM: support vector machine
UPDRS: Unified Parkinson Disease Rating Scale
UPSIT: University of Pennsylvania Smell Identification Test

## Data Availability

The datasets analyzed during this study are available from the Parkinson’s Progression Markers Initiative (PPMI) repository, subject to PPMI data access policies. Selected code and processed data supporting the findings of this study are included in Multimedia Appendix 12. Additional materials can be shared upon reasonable request.
