# Supplementary material for: Prompting and Fine-Tuning Large Language Models for Parkinson Disease Diagnosis: Comparative Evaluation Study Using the PPMI Structured Dataset
Source: JMIR Med Inform. 2026 Jan 15;14:e77561. doi: 10.2196/77561 (PMC12856398; doi:10.2196/77561)
Supplement: Multimedia Appendix 1 [file medinform_v14i1e77561_app1.doc]

Multimedia Appendix 1. Performance and weights of tree-based models (n = 70 variables).

| Model | Accuracy | Recall | Precision | F1-score | Weights |
| --- | --- | --- | --- | --- | --- |
| Random Forest | 0.988 | 0.99 | 0.995 | 0.992 | 0.249 |
| XGBoost | 0.996 | 0.995 | 1 | 0.997 | 0.251 |
| LightGBM | 0.996 | 0.995 | 1 | 0.997 | 0.251 |
| CatBoost | 0.992 | 0.992 | 0.992 | 0.992 | 0.25 |
