# Supplementary material for: Prompting and Fine-Tuning Large Language Models for Parkinson Disease Diagnosis: Comparative Evaluation Study Using the PPMI Structured Dataset
Source: JMIR Med Inform. 2026 Jan 15;14:e77561. doi: 10.2196/77561 (PMC12856398; doi:10.2196/77561)
Supplement: Multimedia Appendix 2 [file medinform_v14i1e77561_app2.doc]

Multimedia Appendix 2. Weighted Mean SHAP Values for the Top 10 Selected Variables.

| No. | Feature Weighted by Importance | Weighted Average SHAP Value |
| --- | --- | --- |
| 1 | updrs3_score | 0.818683906 |
| 2 | con_putamen | 0.487332926 |
| 3 | updrs_totscore | 0.34549527 |
| 4 | updrs2_score | 0.295719421 |
| 5 | lowput_expected | 0.186347613 |
| 6 | upsit_pctl | 0.138839598 |
| 7 | mean_putamen | 0.105246392 |
| 8 | DATSCAN_PUTAMEN_L | 0.092590774 |
| 9 | con_striatum | 0.03530046 |
| 10 | DATSCAN_PUTAMEN_R | 0.031470394 |
