# Supplementary material for: Prompting and Fine-Tuning Large Language Models for Parkinson Disease Diagnosis: Comparative Evaluation Study Using the PPMI Structured Dataset
Source: JMIR Med Inform. 2026 Jan 15;14:e77561. doi: 10.2196/77561 (PMC12856398; doi:10.2196/77561)
Supplement: Multimedia Appendix 3 [file medinform_v14i1e77561_app3.doc]

Multimedia Appendix 3. Prompt Formats by Structure Type and Output Design.^a,b^

| **Prompt** | **Explanation** |
| --- | --- |
| **<|begin_of_text|>**  **<|start_header_id|>system<|end_header_id|** | - No input provided other than Special Tokens.   **-- Special Token** |
| You are a medical expert. Analyze the following test results to determine whether the person likely has Parkinson's disease (Answer: 1) or is a healthy control (Answer: 0). Compare the provided values with typical ranges observed in Parkinson's disease patients and healthy individuals. The values in parentheses represent the weighted average SHAP values of four tree-based models, where higher values indicate greater variable importance. | - Role assignment - Task execution |
| **###** Test Explanations:  1. ******updrs3_score (0.819):****** Represents the MDS-UPDRS Part III score, which evaluates motor function through clinical assessments. A higher score indicates more severe motor symptoms.  2. ******con_putamen (0.487):****** Indicates the dopamine signal intensity ratio between the left and right putamen regions. Parkinson's disease often shows significant asymmetry.  3. ******updrs_totscore (0.345):****** Refers to the total MDS-UPDRS score, reflecting overall severity. A higher score suggests greater disease severity.  4. ******updrs2_score (0.296):****** Evaluates difficulties in daily activities reported by patients. A higher score indicates greater difficulty.  5. ******lowput_expected (0.186):****** Represents the lower expected limit of dopamine signal intensity in the putamen. Below-threshold values suggest dopamine depletion.  6. ******upsit_pctl (0.139):****** Percentile score of the UPSIT test for olfactory function. Lower percentiles suggest smell loss, a biomarker for Parkinson's.  7. ******mean_putamen (0.105):****** Average dopamine signal intensity across both putamen regions, reflecting dopamine deficiency.  8. ******DATSCAN_PUTAMEN_L (0.093):****** Dopamine signal intensity in the left putamen. Asymmetry is a hallmark of Parkinson's.  9. ******con_striatum (0.035):****** Overall dopamine signal intensity in the striatum (including putamen and caudate).  10. ******DATSCAN_PUTAMEN_R (0.031):****** Dopamine signal intensity in the right putamen. Early Parkinson's often shows left-right asymmetry. | - Importance and explanation of PD tests   **-- Mark Down** |
| **###**Example **{i}**:  The following examples demonstrate how to analyze and diagnose based on test data.  Use these patterns to guide your analysis.  1. updrs3_score: **{row['updrs3_score']}**  2. con_putamen: **{row['con_putamen']}**  3. updrs_totscore: **{row['updrs_totscore']}**  4. updrs2_score: **{row['updrs2_score']}**  5. lowput_expected: **{row['lowput_expected']}**  6. upsit_pctl: **{row['upsit_pctl']}**  7. mean_putamen: **{row['mean_putamen']}**  8. DATSCAN_PUTAMEN_L: **{row['DATSCAN_PUTAMEN_L']}**  9. con_striatum: **{row['con_striatum']}**  10. DATSCAN_PUTAMEN_R: **{row['DATSCAN_PUTAMEN_R']}**  Based on the analysis of the provided data, respond with `1` for Parkinson's disease or `0` for healthy control.  ******Final Answer: ****** **{row['COHORT']}**  Please analyze the information provided below carefully. Based on your analysis, respond with `1` if the subject is likely to have Parkinson's disease or `0` if they are healthy. | - Zero-shot: No input provided. - Few-shot: Input is provided.   - One-shot: 1 set per class.   - Two-shot: 2 sets per class. - Blue text: Randomly selected from training values.   **-- Mark Down**  **-- Train value** |
| **<|eot_id|>**  **<|start_header_id|>user<|end_header_id|>** | - No input provided other than Special Tokens.   **-- Special Token** |
| **###** Information of subject:  1. updrs3_score: **{data_point['updrs3_score']}**  2. con_putamen: **{data_point['con_putamen']}**  3. updrs_totscore: **{data_point['updrs_totscore']}**  4. updrs2_score: **{data_point['updrs2_score']}**  5. lowput_expected: **{data_point['lowput_expected']}**  6. upsit_pctl: **{data_point['upsit_pctl']}**  7. mean_putamen: **{data_point['mean_putamen']}**  8. DATSCAN_PUTAMEN_L: **{data_point['DATSCAN_PUTAMEN_L']}**  9. con_striatum: **{data_point['con_striatum']}**  10. DATSCAN_PUTAMEN_R: **{data_point['DATSCAN_PUTAMEN_R']}**  Based on the analysis of the provided data, respond with `1` for Parkinson's disease or `0` for healthy control. Your response should contain only the number `1` or `0`, with no additional text. | - Providing test results - Red text: Test values   **-- Mark Down**  **-- Test value** |
| ******Final Answer: ****** | - Used in few-shot prompting for **binary classification only**. - Response consists of a single numeric value**: 1 (PD) or 0 (HC).** |
| ******Final Answer: ******  [Numeric Answer: 1 or 0]  Reasons for Classification:  1. [First key diagnostic reason]  2. [Second key diagnostic reason]  3. [Third key diagnostic reason] | - Used in **dual-output prompting experiments.** - Model must return both a numeric diagnosis **(1 or 0)** and **three key reasons** supporting the prediction. - Applied to assess both classification performance and explanation consistency |
| **<|eot_id|>**  **<|start_header_id|>assistant<|end_header_id|>** | - No input provided other than Special Tokens.   **-- Special Token** |

^a^This table shows examples of structured prompt formats used for evaluating diagnostic classification and reasoning consistency in large language models. Four format types (PT, MD, ST, MD+ST) were applied under both binary-only and dual-output settings.

^b^Color-coding was used to visually distinguish functional components of the prompts.
 - **Dark red** indicates special tokens (e.g., <|user|>, <|assistant|>),
 - **green** indicates markdown symbols (e.g., ##, **),
 - **blue** represents few-shot training examples, and
 - **red** denotes test-specific input values inserted during inference.
This scheme was applied consistently across all prompt formats.
