# Supplementary material for: Prompting and Fine-Tuning Large Language Models for Parkinson Disease Diagnosis: Comparative Evaluation Study Using the PPMI Structured Dataset
Source: JMIR Med Inform. 2026 Jan 15;14:e77561. doi: 10.2196/77561 (PMC12856398; doi:10.2196/77561)
Supplement: Multimedia Appendix 4 [file medinform_v14i1e77561_app4.doc]

Multimedia Appendix 4. Hyperparameter Configurations for Logistic Regression and Support vector Machine Classifiers.^a-b^

| **Model** | **Solver/ Kernel** | **Regularization** | **Class Weight** | **Additional Notes** |
| --- | --- | --- | --- | --- |
| **Logistic Regression (L2)** | liblinear | L2 penalty (C=1.0) | balanced | No extensive hyperparameter tuning; used as a deterministic baseline. |
| **Support Vector Machine (RBF)** | RBF Kernel | C=1.0,  gamma='scale' | balanced | Kernel-based nonlinear classifier; kept default settings for transparency and reproducibility. |

^a^ Class weights were applied to address the class imbalance between PD and HC in the training set.

^b^ Models were implemented using scikit-learn without additional tuning because the ML baselines were designed to provide transparent and reproducible comparison points rather than optimized performance.
