# Supplementary material for: Prompting and Fine-Tuning Large Language Models for Parkinson Disease Diagnosis: Comparative Evaluation Study Using the PPMI Structured Dataset
Source: JMIR Med Inform. 2026 Jan 15;14:e77561. doi: 10.2196/77561 (PMC12856398; doi:10.2196/77561)
Supplement: Multimedia Appendix 5 [file medinform_v14i1e77561_app5.doc]

**Multimedia Appendix 5. Description of Model Families and API.**

**◆ LLaMA (Meta AI)**

- Meta AI. LLaMA 3: open foundation and instruct models. <https://www.llama.com/models/llama-3/>. Accessed May 6, 2025
- Touvron H, Lavril T, Izacard G, et al. LLaMA: open and efficient foundation language models. arXiv. Published February 27, 2023. doi:10.48550/arXiv.2302.13971

**◆ GPT (OpenAI)**

- OpenAI. GPT-4 models overview. <https://platform.openai.com/docs/models/gpt-4>. Accessed May 6, 2025
- Achiam J, Adler S, Agarwal S, et al. GPT-4 technical report. arXiv. Published March 16, 2023. doi:10.48550/arXiv.2303.08774

**◆ Gemini (Google DeepMind)**

- Google DeepMind. Gemini: our most intelligent AI models. <https://deepmind.google/technologies/gemini/>. Accessed May 6, 2025
- Team G, Anil R, Borgeaud S, et al. Gemini: a family of highly capable multimodal models. arXiv. Published December 18, 2023. doi:10.48550/arXiv.2312.11805

**◆ Claude (Anthropic)**

- Anthropic. Claude’s constitution. <https://www.anthropic.com/news/claudes-constitution>. Accessed May 6, 2025
- Bai Y, Kadavath S, Kundu S, et al. Constitutional AI: harmlessness from AI feedback. arXiv. Published December 15, 2022. doi:10.48550/arXiv.2212.08073
