# Supplementary material for: Prompting and Fine-Tuning Large Language Models for Parkinson Disease Diagnosis: Comparative Evaluation Study Using the PPMI Structured Dataset
Source: JMIR Med Inform. 2026 Jan 15;14:e77561. doi: 10.2196/77561 (PMC12856398; doi:10.2196/77561)
Supplement: Multimedia Appendix 6 [file medinform_v14i1e77561_app6.doc]

Multimedia Appendix 6. Experimental Timeline of Model Evaluations.^a-b^

| Model Family | Model Version  (Official API Model Name) | Prompting / Evaluation Period | External Validation Period |
| --- | --- | --- | --- |
| LLaMA | LLaMA 3.1 8B: Llama-3.1-8B-Instruct LLaMA 3.3 70B: Llama-3.3-70B-Instruct | Few-shot: 2024.12~2025.02  Reasoning: 2025.04~2025.07  Semantic consistency: 2025.06~2025.07 | 2025.06~2025.07 |
| GPT | GPT-4o-mini: 4o-mini (2024-07-18) GPT-4o: 4o (2024-08-06)) | Few-shot: 2024.12~2025.01  Reasoning: 2025.04~2025.07  Fine-tuning: 2025.04.02~2025.07  Semantic consistency: 2025.06~2025.07 |
| Gemini | Gemini 1.5 Flash: gemini-1.5-flash-latest Gemini 1.5 Pro: gemini-1.5-pro-latest | Few-shot: 2025.02~2025.04  Reasoning: 2025.04.04~2025.07  Fine-tuning: 2025.04.03~2025.07  Semantic consistency: 2025.06~2025.07 |
| Claude | Claude 3.5 Sonnet: claude-3.5-sonnet-20241022 | Few-shot: 2025.02~2025.04  Reasoning: 2025.04~2025.07  Semantic consistency: 2025.06~2025.07 |

^a^ Fine-tuning was applied only to lightweight models (GPT-4o-mini, Gemini 1.5 Flash).

Larger models were evaluated without fine-tuning.

^b^ Fine-tuning was not applied to Claude 3.5 Sonnet due to lack of available fine-tuning support at the time of experimentation. In addition, Claude is a commercial model with higher usage costs, which limited extended access.
