# Supplementary material for: Prompting and Fine-Tuning Large Language Models for Parkinson Disease Diagnosis: Comparative Evaluation Study Using the PPMI Structured Dataset
Source: JMIR Med Inform. 2026 Jan 15;14:e77561. doi: 10.2196/77561 (PMC12856398; doi:10.2196/77561)
Supplement: Multimedia Appendix 7 [file medinform_v14i1e77561_app7.doc]

Multimedia Appendix 7. Hardware and Software Configuration.

|  | Workstation 1 | Workstation 2 |
| --- | --- | --- |
| CPU | Intel Xeon w9-3475X | AMD64 Family 23 |
| GPU | NVIDIA RTX A6000 x 3 | NVIDIA RTX A5000 x 3 |
| RAM | 251 GiB | 128 GiB |
| Operating System | Windows 11 Pro | Windows 10 Pro |
