# Supplementary material for: Prompting and Fine-Tuning Large Language Models for Parkinson Disease Diagnosis: Comparative Evaluation Study Using the PPMI Structured Dataset
Source: JMIR Med Inform. 2026 Jan 15;14:e77561. doi: 10.2196/77561 (PMC12856398; doi:10.2196/77561)
Supplement: Multimedia Appendix 8 [file medinform_v14i1e77561_app8.doc]

Multimedia Appendix 8. Summary of Fine-tuning Configurations for Lightweight Language Models.

| Model | Platform | Tuning Method | Training set  (train:val=1,052:186) | Deployment | Notes |
| --- | --- | --- | --- | --- | --- |
| GPT-4o-mini | OpenAI | JSONL fine-tuning | 1,238 | API | - API-based server fine-tuning, job triggered from local |
|  |  |  |  |  | - Auto-managed job (epoch/BS/LR not disclosed) |
| Gemini 1.5 Flash | Google AI Studio | CSV fine-tuning | 500  (only train) | API | - Fine-tuned via web platform, limited to 500 samples |
|  |  |  |  |  | - 1 epoch, batch size 1, learning rate 1 |
