# Supplementary material for: Prompting and Fine-Tuning Large Language Models for Parkinson Disease Diagnosis: Comparative Evaluation Study Using the PPMI Structured Dataset
Source: JMIR Med Inform. 2026 Jan 15;14:e77561. doi: 10.2196/77561 (PMC12856398; doi:10.2196/77561)
Supplement: Multimedia Appendix 9 [file medinform_v14i1e77561_app9.doc]

Multimedia Appendix 9. Diagnostic Performance of Large Language Models in Parkinson’s Disease Classification Using Prompt Engineering and Few-shot Learning (N = 122, 30 Repetitions).

9A. Diagnostic performance of LLaMA 3.1 8B.

| Prompt^a^ | shot | F1-score^b^ | Precision  (macro avg. / PD / HC) | Recall  (macro avg. / PD / HC) | Acc. | Incon.^c^ | Incon. Cases^d^ |
| --- | --- | --- | --- | --- | --- | --- | --- |
| ST | 0 | 0.883 | 0.929 / 0.825 / 1 | 0.862 / 1 / 0.725 | 0.828 | 6/122 | HC #22 (26:4) HC #48 (4:26) HC #49 (14:16) HC #83 (9:21) HC #89 (3:27) HC #122 (25:5) |
|  | 1 | 0.967 | 0.982 / 0.980 / 0.984 | 0.960 / 0.980 / 0.940 | 0.976 | 6/122 | PD #13 (6:24) PD #17 (12:18) HC #23 (25:5) PD #57 (11:19) PD #73 (16:14) HC #103 (25:5) |
|  | 2 | 0.964 | 0.982 / 0.980 / 0.984 | 0.957 / 0.980 / 0.933 | 0.984 | 3/122 | HC #11 (21:9) PD #35 (1:29) PD #57 (6:24) |
|  | 3 | 0.95 | 0.962 / 0.989 / 0.935 | 0.933 / 0.949 / 0.918 | 0.951 | 4/122 | PD #13 (25:5) PD #17 (22:8) PD #57 (23:7) PD #85 (5:25) |
| ST+MD | 0 | 0.857 | 0.903 / 0.846 / 0.960 | 0.833 / 0.924 / 0.742 | 0.853 | 6/122 | HC #22 (24:6) HC #48 (16:14) HC #49 (19:11) HC #83 (14:16) HC #89 (18:12) HC #122 (21:9) |
|  | 1 | 0.934 | 0.956 / 0.942 / 0.969 | 0.924 / 0.980 / 0.867 | 0.975 | 4/122 | HC #11 (19:11) HC #23 (7:23) HC #97 (9:21) HC #113 (22:8) |
|  | 2 | 0.964 | 0.982 / 0.980 / 0.984 | 0.957 / 0.980 / 0.933 | 0.975 | 2/122 | PD #57 (5:25) PD #64 (10:20) |
|  | 3 | 0.967 | 0.982 / 0.980 / 0.984 | 0.960 / 0.980 / 0.940 | 0.992 | 1/122 | PD #73 (13:17) |

9B. Diagnostic performance of LLaMA 3.3 70B.

| Prompt^a^ | shot | F1-score^b^ | Precision  (macro avg. / PD / HC) | Recall  (macro avg. / PD / HC) | Acc. | Incon.^c^ | Incon. Cases^d^ |
| --- | --- | --- | --- | --- | --- | --- | --- |
| PT | 0 | 0.958 | 0.986 / 0.971 / 1 | 0.935 / 1 / 0.870 | 0.975 | 3/122 | HC #23 (1:29) PD #82 (1:29) HC #97 (10:20) |
|  | 1 | 0.899 | 0.944 / 0.917 / 0.971 | 0.893 / 1 / 0.786 | 0.926 | 2/122 | HC #77 (17:13) HC #115 (26:4) |
|  | 2 | 0.967 | 0.990 / 0.980 / 1 | 0.957 / 1 / 0.913 | 0.984 | 3/122 | HC #23 (16:14) HC #68 (24:6) HC #113 (23:7) |
|  | 3 | 0.958 | 0.986 / 0.971 / 1 | 0.935 / 1 / 0.870 | 0.975 | 0/122 | - |
| MD | 0 | 0.947 | 0.961 / 0.980 / 0.943 | 0.939 / 0.980 / 0.898 | 0.967 | 1/122 | PD #111 (21:9) |
|  | 1 | 0.89 | 0.944 / 0.908 / 0.980 | 0.874 / 1 / 0.748 | 0.918 | 1/122 | HC #26 (0:27, none 3) |
|  | 2 | 0.934 | 0.961 / 0.952 / 0.971 | 0.924 / 1 / 0.848 | 0.959 | 0/122 | - |
|  | 3 | 0.934 | 0.961 / 0.952 / 0.971 | 0.924 / 1 / 0.848 | 0.959 | 1/122 | HC #113 (2:28) |
| ST | 0 | 0.973 | 0.990 / 0.980 / 1 | 0.957 / 1 / 0.913 | 0.984 | 1/122 | HC #97 (25:5) |
|  | 1 | 0.89 | 0.944 / 0.908 / 0.980 | 0.874 / 1 / 0.748 | 0.918 | 0/122 | - |
|  | 2 | 0.978 | 0.990 / 0.982 / 0.998 | 0.969 / 1 / 0.938 | 0.992 | 0/122 | - |
|  | 3 | 0.938 | 0.963 / 0.950 / 0.976 | 0.913 / 1 / 0.826 | 0.951 | 0/122 | - |
| ST+MD | 0 | 0.966 | 0.975 / 0.970 / 0.981 | 0.957 / 0.990 / 0.924 | 0.984 | 0/122 | - |
|  | 1 | 0.922 | 0.957 / 0.925 / 0.989 | 0.913 / 1 / 0.826 | 0.934 | 1/122 | HC #26 (17:13) |
|  | 2 | 0.942 | 0.971 / 0.943 / 1 | 0.913 / 1 / 0.826 | 0.967 | 0/122 | - |
|  | 3 | 0.942 | 0.971 / 0.943 / 1 | 0.913 / 1 / 0.826 | 0.967 | 0/122 | - |

9C. Diagnostic performance of GPT-4o-mini.

| Prompt^a^ | shot | F1-score^b^ | Precision  (macro avg. / PD / HC) | Recall  (macro avg. / PD / HC) | Acc. | Incon.^c^ | Incon. Cases^d^ |
| --- | --- | --- | --- | --- | --- | --- | --- |
| PT | 0 | 0.895 | 0.897 / 0.931 / 0.863 | 0.880 / 0.949 / 0.812 | 0.902 | 8/122 | PD #18 (5:25) PD #60 (8:22) PD #70 (28:2) PD #72 (3:27) PD #73 (13:17) HC #86 (27:3) HC #110 (28:2) HC #114 (28:2) |
|  | 1 | 0.887 | 0.913 / 0.892 / 0.935 | 0.875 / 1 / 0.750 | 0.902 | 4/122 | HC #48 (28:2) HC #55 (12:18) HC #103 (1:29) HC #110 (23:7) |
|  | 2 | 0.895 | 0.971 / 0.934 / 1 | 0.913 / 1 / 0.826 | 0.943 | 2/122 | HC #26 (24:6) HC #103 (13:17) |
|  | 3 | 0.908 | 0.978 / 0.943 / 1 | 0.935 / 1 / 0.870 | 0.951 | 2/122 | HC #11 (29:1) HC #103 (29:1) |
| MD | 0 | 0.895 | 0.897 / 0.931 / 0.863 | 0.880 / 0.949 / 0.812 | 0.902 | 5/122 | PD #25 (1:29) PD #73 (3:27) PD #87 (1:29) HC #103 (28:2) HC #110 (16:14) |
|  | 1 | 0.887 | 0.913 / 0.892 / 0.935 | 0.875 / 1 / 0.750 | 0.902 | 3/122 | HC #49 (2:28) HC #103 (2:28) HC #115 (9:21) |
|  | 2 | 0.908 | 0.978 / 0.943 / 1 | 0.935 / 1 / 0.870 | 0.951 | 1/122 | HC #103 (19:11) |
|  | 3 | 0.895 | 0.971 / 0.934 / 1 | 0.913 / 1 / 0.826 | 0.943 | 1/122 | HC #103 (12:18) |

9D. Diagnostic performance of GPT-4o.

| Prompt^a^ | shot | F1-score^b^ | Precision  (macro avg. / PD / HC) | Recall  (macro avg. / PD / HC) | Acc. | Incon.^c^ | Incon. Cases^d^ |
| --- | --- | --- | --- | --- | --- | --- | --- |
| PT | 0 | 0.958 | 0.959 / 0.980 / 0.938 | 0.960 / 0.980 / 0.940 | 0.975 | 5/122 | HC #23 (18:12) PD #64 (2:28) HC #68 (17:13) PD #73 (15:15) PD #80 (6:24) |
|  | 1 | 0.918 | 0.952 / 0.943 / 0.960 | 0.900 / 1 / 0.800 | 0.951 | 3/122 | HC #23 (10:20) HC #26 (12:18) HC #113 (22:9) |
|  | 2 | 0.942 | 0.971 / 0.961 / 0.980 | 0.913 / 1 / 0.826 | 0.967 | 3/122 | HC #26 (24:6) HC #68 (9:21) HC #77 (1:29) |
|  | 3 | 0.942 | 0.971 / 0.961 / 0.980 | 0.913 / 1 / 0.826 | 0.967 | 2/122 | HC #68 (10:20) HC #77 (2:28) |
| MD | 0 | 0.938 | 0.943 / 0.960 / 0.926 | 0.935 / 0.980 / 0.891 | 0.951 | 4/122 | PD #64 (2:28) HC #68 (1:29) PD #82 (9:21) HC #97 (12:18) |
|  | 1 | 0.918 | 0.952 / 0.943 / 0.960 | 0.900 / 1 / 0.800 | 0.959 | 4/122 | HC #23 (12:18) HC #26 (20:10) HC #68 (4:26) HC #113 (28:2) |
|  | 2 | 0.95 | 0.971 / 0.961 / 0.980 | 0.935 / 1 / 0.870 | 0.975 | 2/122 | HC #26 (28:2) HC #68 (17:13) |
|  | 3 | 0.942 | 0.971 / 0.961 / 0.980 | 0.913 / 1 / 0.826 | 0.967 | 3/122 | HC #68 (15:15) HC #77 (6:24) HC #97 (3:27) |

9E. Diagnostic performance of Gemini 1.5 Flash.

| Prompt^a^ | shot | F1-score^b^ | Precision  (macro avg. / PD / HC) | Recall  (macro avg. / PD / HC) | Acc. | Incon.^c^ | Incon. Cases^d^ |
| --- | --- | --- | --- | --- | --- | --- | --- |
| PT | 0 | 0.926 | 0.938 / 0.979 / 0.897 | 0.924 / 0.990 / 0.857 | 0.820 | 1/122 | HC #22 (1:29) |
|  | 1 | 0.877 | 0.917 / 0.884 / 0.950 | 0.865 / 1 / 0.730 | 0.893 | 0/122 | - |
|  | 2 | 0.895 | 0.971 / 0.934 / 1 | 0.913 / 1 / 0.826 | 0.943 | 0/122 | - |
|  | 3 | 0.895 | 0.971 / 0.934 / 1 | 0.913 / 1 / 0.826 | 0.943 | 1/122 | HC #113 (28:2) |
| MD | 0 | 0.87 | 0.932 / 0.936 / 0.928 | 0.870 / 1 / 0.739 | 0.861 | 0/122 | - |
|  | 1 | 0.884 | 0.943 / 0.892 / 0.995 | 0.867 / 1 / 0.734 | 0.902 | 0/122 | - |
|  | 2 | 0.895 | 0.971 / 0.934 / 1 | 0.913 / 1 / 0.826 | 0.943 | 0/122 | - |
|  | 3 | 0.895 | 0.971 / 0.934 / 1 | 0.913 / 1 / 0.826 | 0.943 | 0/122 | - |

9F. Diagnostic performance of Gemini 1.5 Pro.

| Prompt^a^ | shot | F1-score^b^ | Precision  (macro avg. / PD / HC) | Recall  (macro avg. / PD / HC) | Acc. | Incon.^c^ | Incon. Cases^d^ |
| --- | --- | --- | --- | --- | --- | --- | --- |
| PT | 0 | 0.954 | 0.963 / 0.970 / 0.957 | 0.950 / 0.990 / 0.910 | 0.967 | 0/122 | - |
|  | 1 | 0.958 | 0.986 / 0.971 / 1 | 0.935 / 1 / 0.870 | 0.975 | 0/122 | - |
|  | 2 | 0.973 | 0.990 / 0.980 / 1 | 0.957 / 1 / 0.913 | 0.992 | 0/122 | - |
|  | 3 | 0.973 | 0.990 / 0.980 / 1 | 0.957 / 1 / 0.913 | 0.992 | 0/122 | - |
| MD | 0 | 0.939 | 0.950 / 0.960 / 0.940 | 0.935 / 0.980 / 0.891 | 0.951 | 0/122 | - |
|  | 1 | 0.958 | 0.986 / 0.971 / 1 | 0.935 / 1 / 0.870 | 0.975 | 0/122 | - |
|  | 2 | 0.973 | 0.990 / 0.980 / 1 | 0.957 / 1 / 0.913 | 0.992 | 0/122 | - |
|  | 3 | 0.973 | 0.990 / 0.980 / 1 | 0.957 / 1 / 0.913 | 0.992 | 0/122 | - |

9G. Diagnostic performance of Claude 3.5 Sonnet.

| Prompt^a^ | shot | F1-score^b^ | Precision  (macro avg. / PD / HC) | Recall  (macro avg. / PD / HC) | Acc. | Incon.^c^ | Incon. Cases^d^ |
| --- | --- | --- | --- | --- | --- | --- | --- |
| PT | 0 | 0.961 | 0.959 / 0.980 / 0.938 | 0.960 / 0.980 / 0.940 | 0.975 | 2/122 | PD #64 (15:15) PD #104 (29:1) |
|  | 1 | 0.967 | 0.982 / 0.980 / 0.984 | 0.960 / 1 / 0.920 | 0.984 | 1/122 | HC #77 (26:4) |
|  | 2 | 0.967 | 0.982 / 0.980 / 0.984 | 0.960 / 1 / 0.920 | 0.984 | 0/122 | - |
|  | 3 | 0.958 | 0.972 / 0.968 / 0.976 | 0.952 / 1 / 0.904 | 0.975 | 1/122 | HC #97 (25:5) |
| MD | 0 | 0.961 | 0.959 / 0.980 / 0.938 | 0.960 / 0.980 / 0.940 | 0.975 | 1/122 | PD #104 (26:4) |
|  | 1 | 0.967 | 0.982 / 0.980 / 0.984 | 0.960 / 1 / 0.920 | 0.984 | 0/122 | - |
|  | 2 | 0.967 | 0.982 / 0.980 / 0.984 | 0.960 / 1 / 0.920 | 0.984 | 1/122 | PD #26 (2:28) |
|  | 3 | 0.95 | 0.971 / 0.943 / 0.999 | 0.935 / 1 / 0.870 | 0.967 | 0/122 | - |

^a^ Prompt types: PT = plain text; MD = markdown ; ST = Special Token; MD+ST = markdown with special token

^b^ F1-scores represent macro-averaged (macro avg.) values across PD and HC classes.

^c^ Number of participants (n/122) whose predictions were inconsistent at least once across 30 repeated trials.

^d^ Example of inconsistent participants showing the final label (e.g., PD #73) and the number of predicted labels across 30 runs (e.g., 13 : 17 indicates 13 HC and 11 PD predictions).

^e^ Abbreviations: Prompt =Prompt Type; macro avg.=macro-averaged; Acc. = Accuracy; Incon. = Inconsistency
