# Supplementary material for: Prompting and Fine-Tuning Large Language Models for Parkinson Disease Diagnosis: Comparative Evaluation Study Using the PPMI Structured Dataset
Source: JMIR Med Inform. 2026 Jan 15;14:e77561. doi: 10.2196/77561 (PMC12856398; doi:10.2196/77561)
Supplement: Multimedia Appendix 10 [file medinform_v14i1e77561_app10.doc]

Multimedia Appendix 10. Diagnostic Generalization of Large Language Models under Dual-output Prompting on the Test Dataset (N = 122, 30 Repetitions).

10A. Diagnostic performance of LLaMA 3.3 70B.

| Prompt^a^ | shot | F1-score^b^ | Precision (macro avg. / PD / HC) | Recall (macro avg. / PD / HC) | Acc. | Incon.^c^ | Prompt^a^ |
| --- | --- | --- | --- | --- | --- | --- | --- |
| ST | 0 | 0.958 | 0.986 / 0.971 / 1 | 0.935 / 1 / 0.870 | 0.975 | 0/122 | - |
|  | 1 | 0.837 | 0.954 / 0.908 / 1 | 0.783 / 1 / 0.565 | 0.918 | 0/122 | - |
|  | 2 | 0.958 | 0.986 / 0.971 / 1 | 0.935 / 1 / 0.87 | 0.975 | 3/122 | HC #23 (13:17) HC #68 (17:13) HC #77 (24:6) |
|  | 3 | 0.973 | 0.99 / 0.98 / 1 | 0.957 / 1 / 0.913 | 0.984 | 2/122 | HC #23 (29:1)  HC #68 (28:2) |

10B. Diagnostic performance of GPT-4o.

| Prompt^a^ | shot | F1-score^b^ | Precision (macro avg. / PD / HC) | Recall (macro avg. / PD / HC) | Acc. | Incon.^c^ | Prompt^a^ |
| --- | --- | --- | --- | --- | --- | --- | --- |
| PT | 0 | 0.913 | 0.888 / 0.989 / 0.786 | 0.948 / 0.939 / 0.957 | 0.943 | 4/122 | PD #60 (25:5) PD #64 (29:1) PD #76 (4:26) PD #86 (1:29) |
|  | 1 | 0.958 | 0.986 / 0.971 / 1 | 0.935 / 1 / 0.870 | 0.975 | 2/122 | HC #26 (26:4) HC #77 (6:24) |
|  | 2 | 0.958 | 0.986 / 0.971 / 1 | 0.935 / 1 / 0.870 | 0.975 | 2/122 | HC #68 (29:1) HC #77 (2:28) |
|  | 3 | 0.942 | 0.981 / 0.961 / 1 | 0.913 / 1 / 0.826 | 0.967 | 2/122 | HC #26 (23:7) HC #68 (4:26) |

10C. Diagnostic performance of Gemini 1.5 Pro.

| Prompt^a^ | shot | F1-score^b^ | Precision (macro avg. / PD / HC) | Recall (macro avg. / PD / HC) | Acc. | Incon.^c^ | Prompt^a^ |
| --- | --- | --- | --- | --- | --- | --- | --- |
| PT | 0 | 0.922 | 0.910 / 0.979 / 0.840 | 0.936 / 0.959 / 0.913 | 0.951 | 2/122 | HC #23 (1:29) HC #97 (20:10) |
|  | 1 | 0.894 | 0.967 / 0.934 / 1 | 0.848 / 1 / 0.696 | 0.943 | 2/122 | HC #11 (26:4) HC #103 (4:26) |
|  | 2 | 0.942 | 0.981 / 0.961 / 1 | 0.913 / 1 / 0.826 | 0.967 | 1/122 | HC #97 (19:11) |
|  | 3 | 0.958 | 0.986 / 0.971 / 1 | 0.935 / 1 / 0.870 | 0.975 | 1/122 | HC #97 (28:2) |

10D. Diagnostic performance of Claude 3.5 Sonnet.

| Prompt^a^ | shot | F1-score^b^ | Precision (macro avg. / PD / HC) | Recall (macro avg. / PD / HC) | Acc. | Incon.^c^ | Prompt^a^ |
| --- | --- | --- | --- | --- | --- | --- | --- |
| PT | 0 | 0.973 | 0.990 / 0.980 / 1 | 0.957 / 1 / 0.913 | 0.984 | 1/122 | PD #23 (1:29) |
|  | 1 | 0.95 | 0.972 / 0.952 / 0.992 | 0.933 / 1 / 0.867 | 0.959 | 1/122 | HC #23 (28:2) |
|  | 2 | 0.938 | 0.971 / 0.943 / 1 | 0.913 / 1 / 0.826 | 0.951 | 1/122 | HC #11 (14:16) |
|  | 3 | 0.912 | 0.963 / 0.925 / 1 | 0.870 / 1 / 0.739 | 0.934 | 1/122 | HC #30 (5:25) |

^a^ Prompt types: PT = plain text; ST = Special Token

^b^ F1-scores represent macro-averaged (macro avg.) values across PD and HC classes.

^c^ Number of participants (n/122) whose predictions were inconsistent at least once across 30 repeated trials.

^d^ Example of inconsistent participants showing the final label (e.g., PD #73) and the number of predicted labels across 30 runs (e.g., 13 : 17 indicates 13 HC and 11 PD predictions).

^e^ Abbreviations: Prompt =Prompt Type; macro avg.=macro-averaged; Acc. = Accuracy; Incon. = Inconsistency
