# Supplementary material for: Prompting and Fine-Tuning Large Language Models for Parkinson Disease Diagnosis: Comparative Evaluation Study Using the PPMI Structured Dataset
Source: JMIR Med Inform. 2026 Jan 15;14:e77561. doi: 10.2196/77561 (PMC12856398; doi:10.2196/77561)
Supplement: Multimedia Appendix 11 [file medinform_v14i1e77561_app11.doc]

Multimedia Appendix 11. Semantic Consistency of Reasoning Outputs on the Temporal Validation Set under Dual-output Prompting (N = 31).^a,b^

| Model_Few-shot | Mean_Cosine_Similarity | Std_Cosine_Similarity |
| --- | --- | --- |
| LLaMA 3.3 70B_3-shot | 0.980 | ±0.013 |
| GPT-4o_1-shot | 0.973 | ±0.011 |
| Gemini 1.5 Pro_3-shot | 0.962 | ±0.020 |
| Claude 3.5 Sonnet_0-shot | 0.977 | ±0.018 |

^a^ Semantic consistency was assessed based on pairwise cosine similarity among 30 reasoning outputs generated per subject.

^b^ Each value represents the average (mean ± standard deviation) across 31 subjects.
